# Methods for Brain Connectivity Analysis with Applications to Rat Local Field Potential Recordings

**DOI:** 10.3390/e27040328

**Published:** 2025-03-21

**Authors:** Anass B. El-Yaagoubi, Sipan Aslan, Farah Gomawi, Paolo V. Redondo, Sarbojit Roy, Malik S. Sultan, Mara S. Talento, Francine T. Tarrazona, Haibo Wu, Keiland W. Cooper, Norbert J. Fortin, Hernando Ombao

**Affiliations:** 1 Statistics Program, King Abdullah University of Science and Technology (KAUST), Thuwal 23955, Saudi Arabia; 2 Department of Mathematics, Ateneo de Manila University, Quezon City 1108, Philippines; 3 Department of Neurobiology and Behavior, University of California, Irvine, CA 92697, USA

**Keywords:** brain functional connectivity, dependence networks, Granger causality, local field potentials, spectral transfer entropy, topological data analysis, wavelet coherence

## Abstract

Modeling the brain dependence network is central to understanding underlying neural mechanisms such as perception, action, and memory. In this study, we present a broad range of statistical methods for analyzing dependence in a brain network. Leveraging a combination of classical and cutting-edge approaches, we analyze multivariate hippocampal local field potential (LFP) time series data concentrating on the encoding of nonspatial olfactory information in rats. We present the strengths and limitations of each method in capturing neural dynamics and connectivity. Our analysis begins with exploratory techniques, including correlation, partial correlation, spectral matrices, and coherence, to establish foundational connectivity insights. We then investigate advanced methods such as Granger causality (GC), robust canonical coherence analysis, spectral transfer entropy (STE), and wavelet coherence to capture dynamic and nonlinear interactions. Additionally, we investigate the utility of topological data analysis (TDA) to extract multi-scale topological features and explore deep learning-based canonical correlation frameworks for connectivity modeling. This comprehensive approach offers an introduction to the state-of-the-art techniques for the analysis of dependence networks, emphasizing the unique strengths of various methodologies, addressing computational challenges, and paving the way for future research.

## 1. Introduction

Knowledge of the brain structure, function, and mechanisms of underlying neural processes have advanced significantly in recent decades [1,2]. These breakthroughs have been driven by the rapid development of brain imaging techniques, including functional magnetic resonance imaging (fMRI), electroencephalography (EEG), electrocorticography (ECoG), local field potentials (LFPs), and calcium imaging, among others [3]. Equally important are advances in statistical and computational methodologies, enabling the efficient estimation and robust analysis of the complex datasets generated by these imaging techniques [4,5].

Neuroscientists have dedicated immense efforts to understanding both localized brain region function and integration across brain regions during resting state and while responding to external stimuli. It is indeed paramount to estimate and analyze the connectivity patterns between signals, recorded at different brain regions, to uncover the neural mechanisms that underlie perception, action, and cognition [6]. These connectivity patterns not only reveal the strength of connectivity between different brain regions but can also provide information on how disruptions can lead to neurological disorders [7,8,9].

The hippocampus plays a pivotal role in memory formation, spatial navigation, and information processing [10,11,12]. Its importance as a hub for neural connectivity makes it an essential target for studying brain networks. To investigate hippocampal function, researchers often rely on animal models (e.g., macaques and rats) whose brain structures bear a strong resemblance to those of humans [13]. These models allow for precise and invasive experimental designs that are considered unethical in human studies.

This paper explores some advanced methods commonly used to analyze brain networks. We apply them to hippocampal LFP data from the CA1 region to investigate the encoding of nonspatial olfactory information in rats. Our aim is to introduce these methods to readers while highlighting the insights they can offer into neural connectivity. We will present and discuss the differences between classical methods and state-of-the-art approaches in modeling brain connectivity. Beyond showcasing the strengths, we also discuss their limitations and identify areas for potential future improvement. By presenting a wide set of techniques, we provide readers with tools to analyze different aspects of brain connectivity, offering diverse perspectives and insights into complex neural systems.

The structure of this paper is organized as follows: Section 2 introduces the dataset and provides formal definitions of foundational concepts such as correlation, partial correlation, and coherence, which are essential for understanding basic connectivity patterns. Section 3 details robust canonical coherence, a method for assessing more complex interdependencies. Section 4 presents a hybrid approach combining Spectral Dynamic Principal Component Analysis (sDPCA) with Granger causality (GC) to analyze directional influences among specific channels and mitigate confounding effects from the broader network. Section 5 explores spectral transfer entropy (STE), an information-theoretic method that examines frequency-specific influence and information flow in the brain. Section 6 discusses wavelet coherence, which captures dynamic and nonlinear interactions between brain regions. Section 7 introduces Persistence Homology (PH), a topological method that avoids the need for thresholding in weighted networks and extracts multi-scale connectivity patterns, thus detecting higher-order interactions. Section 8 summarizes and discusses the strengths and limitations of the methods presented, emphasizing their potential for enhancing brain connectivity analysis and identifying promising directions for future research. Finally, Section 9 offers concluding remarks.

## 2. Exploratory Data Analysis

In this section, we turn our attention to the experimental data that underpin our analyses. The dataset consists of local field potential (LFP) recordings from the CA1 region of the hippocampus in rats performing a nonspatial sequence memory task, a paradigm chosen for its strong behavioral parallels between rats and humans. As detailed in Allen et al. [14], LFP signals were recorded from five male Long–Evans rats. The animals were individually housed, with water access controlled during weekdays (serving as a reward in odor memory tasks).

**Figure 1:**
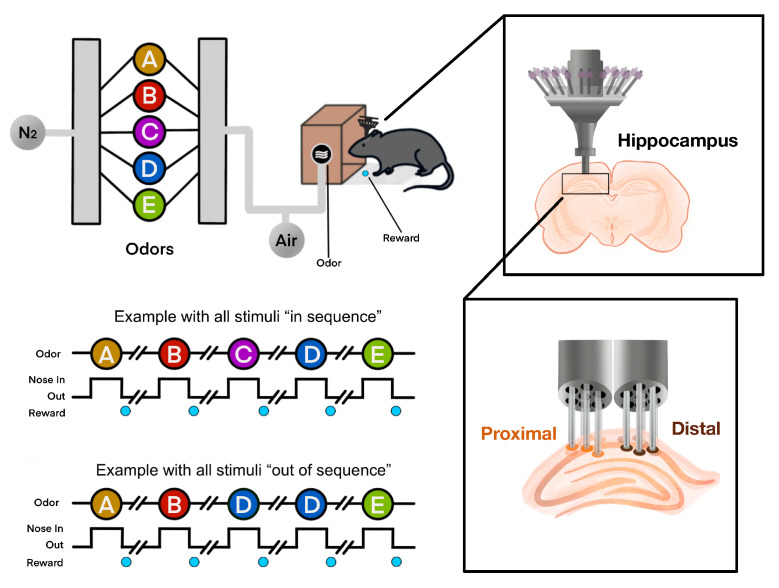
Experimental setup, including the trial scheme for in-sequence and out-of-sequence odor presentations and the positioning of tetrodes in the hippocampal CA1 region.

The experimental protocol involved a nonspatial sequence memory task, in which rats were required to memorize and recognize a fixed sequence of five odors: lemon (A), anise (B), rum (C), vanilla (D), and banana (E). Rats underwent an incremental training protocol over 6–8 weeks. Initially, naive rats were trained to nosepoke and maintain their nose in the odor port for a water reward. The required nosepoke duration was gradually increased from 50 ms in 15 ms steps until reaching 1.2 s, with a criterion of 80% correct responses over three consecutive sessions (100–200 nosepokes per session). Subsequently, the animals were habituated to odor presentations, first with a single odor (Odor A) and then with a two-odor sequence (Odors A and B), both requiring a 1.2 s nosepoke for a reward. Once performance was stabilized, the rats were trained to discriminate between in-sequence and out-of-sequence presentations, starting with a two-item sequence (e.g., “AB” for in-sequence versus “AA” for out-of-sequence) and progressing to sequences of three, four, and finally five odors. After achieving criterion performance on the five-item sequence, the rats underwent microdrive implantation surgery for subsequent electrophysiological recordings. The odors were delivered through a single odor port as described in Figure 1, with each session featuring odors presented either in the correct sequential order or with at least one item out-of-sequence.

In this study, the dataset is organized into four-second trials, with odor presentation occurring at the midpoint (two seconds) and initiated by a nosepoke. LFP signals were recorded at a sampling rate of 1000 Hz from five rats using a microdrive equipped with approximately 20–22 tetrodes per rat, each positioned in either the proximal or distal region of the CA1 layer. On average, each rat completed between 170 and 300 trials (approximately 170–260 in-sequence and 20–45 out-of-sequence trials). Notably, the ‘Barat’ rat demonstrated the highest accuracy in recognizing in-sequence odors, while ‘Superchris’ excelled in identifying out-of-sequence presentations. Detailed variations in the number of tetrodes and trials across subjects are provided in Table 1.

### 2.1. Classical Dependence Measures

For the remainder of this manuscript, we adopt the following unified notation to facilitate the presentation of analytical methods. Consider LFP signals measured over time from *P* tetrodes. Let $X_{p,t}$ represent the LFP signal recorded from the *p*-th tetrode at time *t*, where p=1,…,P. For multiple trials of the same experiment, we use the superscript notation $X_{p,t}^{\left(r\right)}$ to denote the *r*-th trial. For clarity, we refer to tetrodes 1,2,…,P as T1,T2,…,TP, respectively.

Functional connectivity (FC) refers to the statistical association between neurophysiological events measured across various scales, microscale (individual neurons), mesoscale (neuronal populations), and macroscale (brain regions) [15]. In the context of brain connectivity analysis, FC is almost always measured using Pearson correlations [16]. In this study, we focus on LFP signals, which serve as mesoscale measurements that capture the collective activity of multiple neurons. Specifically, we investigate FC between tetrodes using correlation and partial correlation as defined below.

Consider the LFP signals recorded during the *r*-th trial from two tetrodes *p* and *q*, denoted by $\left\{X_{p,t}^{\left(r\right)}\right\}$ and $\left\{X_{q,t}^{\left(r\right)}\right\}$. For this section, we assume the LFP during trial *r*$\left\{X_{p,t}^{\left(r\right)}\right\}$ and $\left\{X_{q,t}^{\left(r\right)}\right\}$ to be the zero-mean second-order stationary time series (see [17] for definition). Then, the *cross-covariance* between $X_{p,t}^{\left(r\right)}$ and $X_{q,t}^{\left(r\right)}$ at time delay *k* is written as

(1) $$\sigma_{pq}^{\left(r\right)}\left(k\right)=\mathrm{Cov}\left[X_{p,t}^{\left(r\right)},X_{q,t+k}^{\left(r\right)}\right]\;=\;E\left[X_{p,t}^{\left(r\right)}\;X_{q,t+k}^{\left(r\right)}\right].$$

Given *P* tetrodes in the system, all pairwise covariances can be compactly written as a P×P cross-covariance matrix at lag *k*, denoted as $\Sigma^{\left(r\right)}\left(k\right)$, i.e.,

(2) $$\Sigma^{\left(r\right)}\left(k\right)\;=\;\left[\begin{array}{cccc} \sigma_{11}^{\left(r\right)}\left(k\right) & \sigma_{12}^{\left(r\right)}\left(k\right) & \cdots & \sigma_{1P}^{\left(r\right)}\left(k\right)\\ \sigma_{21}^{\left(r\right)}\left(k\right) & \sigma_{22}^{\left(r\right)}\left(k\right) & \cdots & \sigma_{2P}^{\left(r\right)}\left(k\right)\\ \vdots & \vdots & \ddots & \vdots \\ \sigma_{P1}^{\left(r\right)}\left(k\right) & \sigma_{P2}^{\left(r\right)}\left(k\right) & \cdots & \sigma_{PP}^{\left(r\right)}\left(k\right) \end{array}\right]$$

Although cross-covariance quantifies the dependence between two time series, it is often difficult to interpret because its magnitude depends on the scale (level of variability) of the data. Thus, it is more common to use its scaled version, called *cross-correlation* or simply correlation, which takes values in the interval [1,1]. This is more useful especially when comparing strengths of connectivity across different tetrode pairs. More precisely, the correlation between $X_{p,t}^{\left(r\right)}$ and $X_{q,t+k}^{\left(r\right)}$ is defined as

$$\rho_{pq}^{\left(r\right)}\left(k\right)=\frac{\mathrm{Cov}\left[X_{p,t}^{\left(r\right)},X_{q,t+k}^{\left(r\right)}\right]}{\sqrt{\mathrm{Var}\left[X_{p,t}^{\left(r\right)}\right]\mathrm{Var}\left[X_{q,t}^{\left(r\right)}\right]}}=\frac{\sigma_{pq}^{\left(r\right)}\left(k\right)}{\sqrt{\sigma_{pp}^{\left(r\right)}\left(0\right)\cdot \sigma_{qq}^{\left(r\right)}\left(0\right)}}.$$

The correlation index $\rho_{pq}^{\left(r\right)}\left(k\right)$ measures the linear association between the signals $X_{p,t}^{\left(r\right)}$ and $X_{q,t+k}^{\left(r\right)}$. Moreover, when the LFPs have a normal distribution, $\rho_{pq}^{\left(r\right)}\left(k\right)=0$ implies unconditional independence between them. However, one limitation is that it may include confounding variables that influence the interaction between $X_{p,t}^{\left(r\right)}$ and $X_{q,t}^{\left(r\right)}$, e.g., another signal from the same system, say $X_{v,t}^{\left(r\right)}$. Thus, an alternative approach is to quantify the direct dependence between a pair of signals after *taking into account* the contributions of other components in the brain network. This is offered by the *partial correlation* measure, which we define below.

Define v to be the set of P−2 tetrodes excluding the *p*-th and *q*-th tetrodes, and $X_{v,t}^{\left(r\right)}$ to be the multivariate time series recorded during the *r*-th trial from all tetrodes in v. Note that $X_{p,t}^{\left(r\right)}$ and $X_{q,t}^{\left(r\right)}$ are excluded in $X_{v,t}^{\left(r\right)}$. Consider the variance–covariance matrix at lag 0, which can be derived from Equation (2), and denote it by $\Sigma^{\left(r\right)}=\Sigma^{\left(r\right)}\left(0\right)$. The precision matrix, denoted by $\Theta^{\left(r\right)}$, is the inverse of $\Sigma^{\left(r\right)}$, i.e.,

$$\Theta^{\left(r\right)}={\left[\Sigma^{\left(r\right)}\right]}^{-1}\;=\;\left[\begin{array}{cccc} \Theta_{11}^{\left(r\right)} & \Theta_{12}^{\left(r\right)} & \cdots & \Theta_{1P}^{\left(r\right)}\\ \Theta_{21}^{\left(r\right)} & \Theta_{22}^{\left(r\right)} & \cdots & \Theta_{2P}^{\left(r\right)}\\ \vdots & \vdots & \ddots & \vdots \\ \Theta_{P1}^{\left(r\right)} & \Theta_{P2}^{\left(r\right)} & \cdots & \Theta_{PP}^{\left(r\right)} \end{array}\right]$$

Then, the partial correlation between two tetrodes $X_{p,t}$ and $X_{q,t}$, after removing the linear contributions of the remaining tetrodes in the system $X_{v,t}$, is defined to be

$$\rho_{pq|v}^{\left(r\right)}=-\frac{\Theta_{pq}^{\left(r\right)}}{\sqrt{\Theta_{pp}^{\left(r\right)}\cdot \Theta_{qq}^{\left(r\right)}}}.$$

The quantity $\Theta_{pq}^{\left(r\right)}$ represents the element in the *p*-th row, *q*-th column of the precision matrix, which in practice, may be obtained as the inverse of an estimated variance–covariance matrix. A caveat, however, is that the covariance matrix should be positive definite (and hence non-singular) for the precision matrix to exist. In cases of perfect collinearity between at least one pair of signals, the covariance matrix is singular, preventing the signals from being de-confounded.

#### 2.1.1. Permutation Test

For a given odor, consider the correlation between the LFP signals, recorded during the *r*-th trial, from tetrodes *p* and *q* and denote it by $\rho_{pq}^{\left(r\right)}$. Here, we compare the two groups of trials (in-sequence vs. out-of-sequence). Denote by $\left\{\rho_{pq}^{\left(1\right)},\rho_{pq}^{\left(2\right)}\right\}$ and $\left\{\nu_{pq}^{\left(1\right)},\nu_{pq}^{\left(2\right)}\right\}$ the respective true means of the correlations and variances across the entire distribution all possible realizations of in-sequence and out-of-sequence trials. Our goal is to determine whether there are differences in the mean correlations between in vs. out-of sequence states, e.g., $n_{1}$ in-sequence correct trials and $n_{2}$ out-of-sequence correct trials with zero time delay (k=0). Hence, for a given {p,q}-tetrode pair, we wish to test the following hypothesis:

$$\begin{array}{c} H_{0}:\rho_{pq}^{\left(1\right)}=\rho_{pq}^{\left(2\right)}\;\;\mathrm{vs}.\;\;H_{a}:\rho_{pq}^{\left(1\right)}\neq \rho_{pq}^{\left(2\right)}. \end{array}$$

We consider the test statistic

$$T_{\mathrm{stat}}=\frac{{\hat{\rho }}_{pq}^{\left(1\right)}-{\hat{\rho }}_{pq}^{\left(2\right)}}{\sqrt{\frac{{\hat{\nu }}_{pq}^{\left(1\right)}}{n_{1}}+\frac{{\hat{\nu }}_{pq}^{\left(2\right)}}{n_{2}}}},$$

where $\left\{{\hat{\rho }}_{pq}^{\left(1\right)},{\hat{\rho }}_{pq}^{\left(2\right)}\right\}$ and $\left\{{\hat{\nu }}_{pq}^{\left(1\right)},{\hat{\nu }}_{pq}^{\left(2\right)}\right\}$ are the group *sample* averages and group *sample* variances, respectively, of the correlations. Given the observed data, let $t_{\mathrm{obs}}$ denote the calculated value of the test statistics $T_{\mathrm{stat}}$. As a decision rule, we reject the null hypothesis $H_{0}$ if the *p*-value, i.e., $p=\mathrm{Pr}(|T_{\mathrm{stat}}|\geq |t_{\mathrm{obs}}||H_{0}\;\mathrm{is}\;\mathrm{true})$, is less than the significance level α.

One approach is to empirically derive the unknown distribution of $T_{\mathrm{stat}}$ under the null and thus estimate the *p*-value through a permutation testing scheme. Under the null hypothesis, the correlations $\rho_{pq}^{\left(r\right)}$ from all correct trials, whether in-sequence or out-of-sequence, come from the same distribution. Such an assumption allows the reassignment or relabeling of the correlations as in-sequence or out-of-sequence, which corresponds to one permutation. In an iterative manner, several permutations of labels for the observed correlations are obtained, and for each permutation, a $t_{\mathrm{obs}}$ is calculated. The collection of $t_{\mathrm{obs}}$ values comprises the empirical null distribution of $T_{\mathrm{stat}}$, from which we obtain the *p*-value for the two-sample *t*-test.

#### 2.1.2. Correlation Analysis of the LFP Dataset

**Figure 2:**
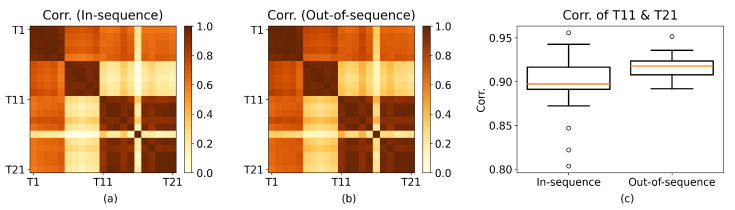
The mean correlation matrices of LFP recordings from (**a**) in-sequence and (**b**) out-of-sequence trials performed with vanilla. The boxplot in (**c**) visualizes the distributions of the correlations of LFP recordings between T11 and T21.

We now implement the permutation test (discussed above) on the correlations of LFP signals from the rat named “Superchris” for in-sequence and out-of-sequence trials of all combinations of odors and pairs of tetrodes (Figure 2). In particular, we examine trials where the odor presented is *vanilla*. For recordings from T11 and T21, there is a significant difference between the mean correlations of the in-sequence trials and the out-of-sequence trials at α=0.05, with $t_{\mathrm{obs}}\approx -2.3148$ and p≈0.0342.

**Figure 3:**
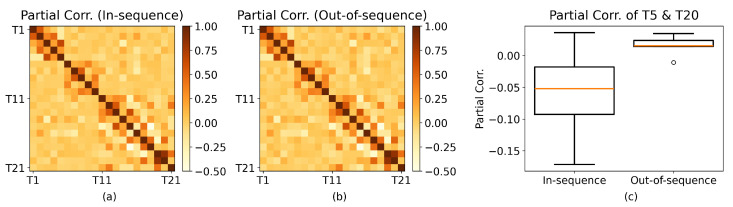
The mean partial correlation matrices of LFP recordings from (**a**) in-sequence and (**b**) out-of-sequence trials performed with rum. The boxplot in (**c**) visualizes the distributions of the correlations of LFP recordings between T5 and T20.

The same analysis can be performed for partial correlation (Figure 3), accounting for the confounding variable results in sparse correlation matrices. For rum and tetrode-pair T5–T20, mean partial correlations between in-sequence and out-of-sequence trials have a significant difference ($t_{\mathrm{stat}}\approx -6.8059;p\approx 0.0007;\alpha =0.05$), revealing a change in interaction depending on the accuracy of the odor sequence.

### 2.2. Spectral Dependence Measures

Correlation and partial correlation are simple yet effective measures for capturing the linear dependence between signals in the time domain. In contrast, assessing synchronization in the frequency domain provides a more detailed understanding of the oscillatory dynamics that drive neural interactions. Coherence analysis, the frequency-domain counterpart of correlation, has proven highly effective in evaluating brain connectivity by yielding results that are directly interpretable in terms of frequency components [18].

Let $X_{t}={\left[X_{1,t}^{\left(r\right)},\ldots ,X_{P,t}^{\left(r\right)}\right]}^{⊤}$ be a *P*-dimensional second-order stationary time series, meaning that its mean vector,

$$E\left[X_{t}\right]=\mu_{X},$$

remains constant over time, and its covariance matrix,

$$\mathrm{Cov}\left(X_{t},X_{t+k}\right)=\Sigma \left(k\right),$$

depends only on the lag *k* rather than the specific time index *t*. In addition, we assume that the elements of Σ(k) are absolutely summable:

$$\sum_{k=-\infty }^{\infty }\left|\sigma_{pq}\left(k\right)\right|<\infty \;\forall \;p,q.$$

These conditions ensure the existence of a well-defined spectral matrix for $X_{t}$.

The essence of the spectral analysis is to represent brain signals as a superposition of oscillatory components across various frequency bands. This is achieved by decomposing the signal into complex exponentials, where

$$e^{i2\pi \omega t}=\cos\left(2\pi \omega t\right)+i\sin\left(2\pi \omega t\right)$$

serves as the fundamental building block. This idea is formalized in the *Cramér representation*:

(3) $$X_{t}=\int_{-\pi }^{\pi }e^{i2\pi \omega t}\;\mathrm{dZ}\left(\omega \right),$$

with dZ(ω) denoting a zero-mean, orthogonal increment process. This representation holds under the aforementioned stationarity conditions.

The spectrum of an individual component $X_{p,t}$ is defined as the Fourier transform of its autocovariance function:

(4) $$S_{pp}^{\left(r\right)}\left(\omega \right)=\sum_{k=-\infty }^{+\infty }\sigma_{pp}^{\left(r\right)}\left(k\right)\;e^{-i2\pi \omega k},$$

and the cross-spectrum between components $X_{p,t}$ and $X_{q,t}$ is similarly given by the Fourier transform of their cross-covariance function:

(5) $$S_{pq}^{\left(r\right)}\left(\omega \right)=\sum_{k=-\infty }^{+\infty }\sigma_{pq}^{\left(r\right)}\left(k\right)\;e^{-i2\pi \omega k}.$$

Collecting all auto- and cross-spectral quantities from the spectral matrix

(6) $$S^{\left(r\right)}\left(\omega \right)\;=\;\left[\begin{array}{cccc} S_{11}^{\left(r\right)}\left(\omega \right) & S_{12}^{\left(r\right)}\left(\omega \right) & \cdots & S_{1P}^{\left(r\right)}\left(\omega \right)\\ S_{21}^{\left(r\right)}\left(\omega \right) & S_{22}^{\left(r\right)}\left(\omega \right) & \cdots & S_{2P}^{\left(r\right)}\left(\omega \right)\\ \vdots & \vdots & \ddots & \vdots \\ S_{P1}^{\left(r\right)}\left(\omega \right) & S_{P2}^{\left(r\right)}\left(\omega \right) & \cdots & S_{PP}^{\left(r\right)}\left(\omega \right). \end{array}\right]$$

The spectral matrix can provide insight into the spectral power distribution of the signals. However, by examining dominant specific frequency bands that are present in the signals, one can develop a better understanding of the brain connectivity and mental state of a subject. Studies have demonstrated that the five traditional frequency bands are associated with cognitive states [19] and can also be used as potential biomarkers for neurological diseases (e.g., autism, and attention deficit-hyperactivity disorder (ADHD)) [20]. These frequency bands, which are defined below, can be adapted in the analysis of LFP signals.

**Figure 4:**
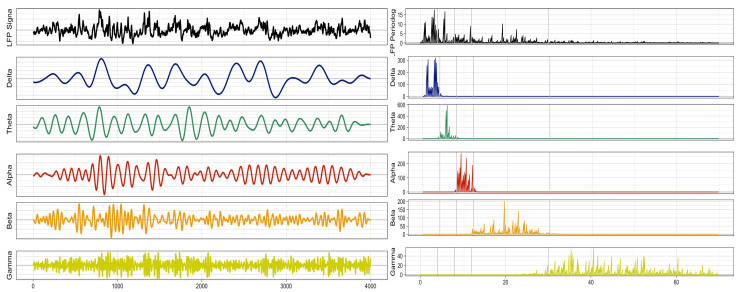
A sample LFP signal decomposition and spectral power.

The most common frequency bands of interest in EEG and LFP analysis are $\Omega_{1}\in \left(0.5,4\right)$ Hertz, $\Omega_{2}\in \left(4,8\right)$ Hertz, $\Omega_{3}\in \left(8,12\right)$ Hertz, $\Omega_{4}\in \left(12,30\right)$ Hertz, and $\Omega_{5}\in \left(30,50\right)$ Hertz. There is a 1-1 mapping between the frequency band in the generic interval (0,0.50) and the bands in practical EEG/LFP, which is defined as follows. Let *s* be the sampling rate, and let $\left(\omega_{L},\omega_{H}\right)\in \left(0,0.50\right)$ be the generic band of interest. This corresponds to $\Omega =(s.\omega_{L},s.\omega_{H})$. Using these bands of interest, we can decompose the observed LFP to be

$$X_{t}=a_{1}X_{t}^{\Omega_{1}}+a_{2}X_{t}^{\Omega_{2}}+a_{3}X_{t}^{\Omega_{3}}+a_{4}X_{t}^{\Omega_{4}}+a_{5}X_{t}^{\Omega_{5}},$$

where $a_{j},\;j=1,\ldots ,5$ are weights associated to the contribution of each frequency band in the signal. Additionally, $X_{t}^{\Omega_{j}}$ can be derived from the observed signal via linear filtering (e.g., Butterworth filter)

(7) $$X_{t}^{\Omega_{j}}=\sum_{k=-\infty }^{\infty }h\left(k\right)X_{t-k}$$

where the filter {h(k)} is selected so that the power of $X_{t}^{\Omega_{j}}$ is concentrated at the frequency band $\Omega_{j}$. The filtered decomposition for a sample LFP signal from a trial is shown in Figure 4, at which the left-hand side showcases the spectral power and the right is the decomposed signals for each of the signals.

Dependence between tetrodes can be characterized via *coherence* which is a frequency domain measure of linear correlation between two signals of the same frequency band $\Omega_{j}$. For tetrodes *p* and *q*, the coherence at frequency ω is defined as

(8) $$C_{pq}\left(\Omega_{j}\right)\left(\omega \right)=\frac{{\left|S_{pq}\left(\Omega_{j}\right)\left(\omega \right)\right|}^{2}}{S_{pp}\left(\Omega_{j}\right)\left(\omega \right)\;S_{qq}\left(\Omega_{j}\right)\left(\omega \right)}\;\;;\;i=1,2,3,4,5,$$

where $S_{pp}\left(\Omega_{j}\right)$ and $S_{pq}\left(\Omega_{j}\right)$ are the auto-spectrum and cross-spectrum at band $\Omega_{j}$. The values of coherence lie between 0 and 1, with 0 indicating that there is no linear correlation at that frequency and 1 indicating a perfect linear relationship at that frequency.

**Figure 5:**
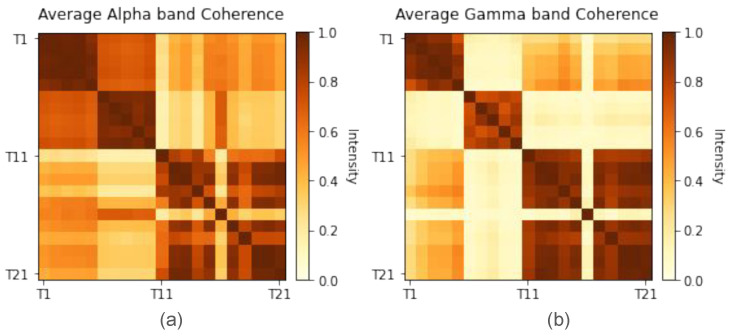
The average pairwise coherence for the (**a**) alpha and (**b**) gamma frequency bands.

It is of interest to see that the resulting coherence is also clustered within the same tetrodes as in Figure 2. Nonetheless, Figure 5 shows that there is a difference in the intensity of synchronization between tetrode signals at the alpha and gamma frequency bands. It appears that even though the alpha band has lower within-cluster coherence, it shows overall higher coherence in many tetrodes further from the diagonal. On the other hand, the gamma frequency band shows clearer coherence intensities; the clusters near the diagonal have high coherence, and clusters far from the diagonal have low coherence.

Coherence analysis can be a useful tool for looking at brain connectivity. However, it comes with the assumption of the stationarity of the time series signals, which can be the case for a short time frame but cannot hold for a longer time, as many real-world signals are non-stationary. Additionally, the coherence of the frequency domain does not indicate the temporal information when the signal pairs are coherent, and coherence analysis can require averaging over time windows (trials) in order to estimate the spectral and cross-spectral power. Consequently, coherence gives a global frequency relationship and is sensitive to noise, as it can inflate or deflate the coherence between signals.

Nonetheless, in Section 6, wavelet analysis, which does not require stationarity for the time series, is discussed as another spectral domain analysis method. The wavelets are less sensitive to noise and can capture multi-scale relationships. Additionally, looking at pairwise analysis can result in redundancies in real-life applications. Therefore, in Section 3, coherence is used to obtain connectivity measures between a cluster of tetrodes from major brain regions.

## 3. Robust Canonical Coherence

**Figure 6:**
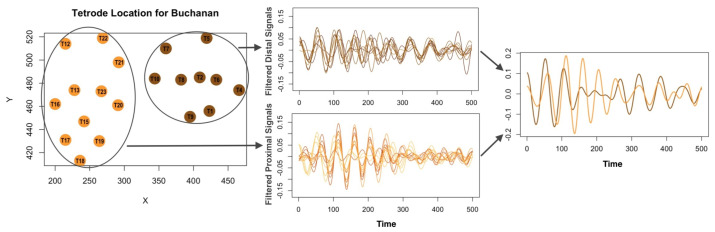
Spatial arrangement of tetrodes, filtered signals, and their weighted combinations. The spatial arrangement of 20 tetrodes from Buchanan is shown in *x*-*y* coordinates, divided into proximal (orange) and distal (brown) sections (**left**). Filtered signals within the frequency range Ω=(12–30)Hz from the 20 tetrodes are displayed (**middle**). Weighted linear combinations of the signals are computed separately for each section (**right**).

A common approach in network analysis focuses on evaluating pairwise connectivity between channels. However, in many real-world scenarios, assessing dependencies between entire regions rather than individual channels often provides deeper insights. To illustrate this, we plot the LFP signals from 20 tetrodes of Buchanan in Figure 6. The left panel shows the location of these tetrodes that are grouped according to their spatial orientation. The middle panel demonstrates that signals within each region or section exhibit a higher degree of synchrony. We then *combine* the signals within each region (the right panel). A natural approach to summarizing the dependency between the regions is to consider the correlation between linear combinations of the signals. The method of obtaining the optimal linear combination that maximizes this correlation is referred to as the canonical coherence analysis. We begin with formally defining the canonical coherence.

### 3.1. Canonical Coherence

Let ${\left\{Z_{t}\right\}}_{t=1}^{T}$ be a *d*-dimensional weakly stationary time series, where $Z_{t}^{⊤}=\left[X_{t}^{⊤},Y_{t}^{⊤}\right]$, for t=1,…,T with d=P+Q. Recall the definition of the spectral density matrix (SDM) in Equation (6) and note that the SDM of $Z_{t}$ can be expressed as

$$S_{ZZ}\left(\omega \right)=\left(\begin{array}{cc} S_{XX}\left(\omega \right) & S_{XY}\left(\omega \right)\\ S_{YX}\left(\omega \right) & S_{YY}\left(\omega \right) \end{array}\right),$$

where $S_{XX}\left(\omega \right)$ and $S_{YY}\left(\omega \right)$ are the auto-SDM, while $S_{XY}\left(\omega \right)$ and $S_{YX}\left(\omega \right)$ are the cross-SDM of $X_{t}$ and $Y_{t}$ such that $S_{XY}\left(\omega \right)=S_{YX}^{H}\left(\omega \right)$, where H denotes the conjugate-transpose operator. Recall the *Cramér representation* in Equation (3). Canonical coherence analysis at ω-oscillation finds vectors $a_{\omega }\in C^{P}$ and $b_{\omega }\in C^{Q}$ that maximize the coherence (see Equation (8)) between $a_{\omega }^{H}X\left(d\omega \right)$ and $b_{\omega }^{H}Y\left(d\omega \right)$. The canonical coherence ϕ(ω) as defined in [21] is given by

(9) $$\phi \left(\omega \right)=\max_{a_{\omega },b_{\omega }}{\left|a_{\omega }^{H}S_{XY}\left(\omega \right)b_{\omega }\right|}^{2}\;\mathrm{such}\;\mathrm{that}\;a_{\omega }^{H}S_{XX}\left(\omega \right)a_{\omega }=b_{\omega }^{H}S_{YY}\left(\omega \right)b_{\omega }=1.$$

This approach, however, is limited to capturing the linear dependence between $X_{t}$ and $Y_{t}$ at a singleton frequency ω. One approach to mitigate this limitation is to consider the canonical *band*-coherence (CBC) (see [22]) that is defined using band-specific filtered signals introduced in Section 2.2. In the following subsection, we first introduce CBC and then present a robust procedure for estimating CBC for a given frequency band Ω.

### 3.2. Canonical Band Coherence

First, we recall the definition of a filtered series as given in Equation (7). Let $Z_{t}^{\Omega }={\left[Z_{1,t}^{\Omega },\ldots ,Z_{d,t}^{\Omega }\right]}^{⊤}={\left[X_{1,t}^{\Omega },\ldots ,X_{P,t}^{\Omega },Y_{1,t}^{\Omega },\ldots ,Y_{Q,t}^{\Omega }\right]}^{⊤}$ denote the filtered signals corresponding to the frequency band Ω. Here, $Z_{j,t}^{\Omega }$ represents the *j*-th channel, with j=1,…,d, and t=1,…,T. Let $\Sigma_{Z}^{\Omega }\left(h\right)$ denote the covariance between the filtered signals $Z_{t-h}^{\Omega }$ and $Z_{t}^{\Omega }$, i.e., $\Sigma_{Z}^{\Omega }\left(h\right)=\mathrm{Cov}\left(Z_{t-h}^{\Omega },Z_{t}^{\Omega }\right)$. Subsequently, we write $\Sigma_{Z}^{\Omega }\left(h\right)$ as

$$\Sigma_{Z}^{\Omega }\left(h\right)=\left(\begin{array}{cc} \mathrm{Cov}(X_{t-h}^{\Omega },X_{t}^{\Omega }) & \mathrm{Cov}(X_{t-h}^{\Omega },Y_{t}^{\Omega })\\ \mathrm{Cov}(Y_{t-h}^{\Omega },X_{t}^{\Omega }) & \mathrm{Cov}(Y_{t-h}^{\Omega },Y_{t}^{\Omega }) \end{array}\right)=\left(\begin{array}{cc} \Sigma_{XX}^{\Omega }\left(h\right) & \Sigma_{XY}^{\Omega }\left(h\right)\\ \Sigma_{YX}^{\Omega }\left(h\right) & \Sigma_{YY}^{\Omega }\left(h\right) \end{array}\right).$$

The authors in [22] defined the CBC between $X_{t-h}$ and $Y_{t}$ for a frequency band Ω as the maximum squared correlation between their linear combinations:

$$\phi \left(\Omega \right)=\max_{u,v,h}{\left|\mathrm{Cor}\left(u^{⊤}X_{t-h}^{\Omega },v^{⊤}Y_{t}^{\Omega }\right)\right|}^{2}=\max_{u,v,h}\frac{{\left|\mathrm{Cov}\left(u^{⊤}X_{t-h}^{\Omega },v^{⊤}Y_{t}^{\Omega }\right)\right|}^{2}}{V\left(u^{⊤}X_{t-h}^{\Omega }\right)V\left(v^{⊤}Y_{t}^{\Omega }\right)}.$$

The CBC denoted by ϕ(Ω) can be further expressed as

(10) $$\phi \left(\Omega \right)=\max_{u,v,h}{\left|u^{⊤}\Sigma_{XY}^{\Omega }\left(h\right)v\right|}^{2}\;\mathrm{such}\;\mathrm{that}\;u^{⊤}\Sigma_{XX}^{\Omega }\left(0\right)u=v^{⊤}\Sigma_{YY}^{\Omega }\left(0\right)v=1.$$

Let $u_{\Omega }$, $v_{\Omega }$ be the vectors and $h_{\Omega }$ be the lag that maximize Equation (10). These vectors $u_{\Omega }$ and $v_{\Omega }$ are called *canonical directions*, and $h_{\Omega }$ is referred to as the *canonical lag* at frequency band Ω. The magnitude of elements of the canonical directions provides a measure of each channel’s contribution to the CBC between $X_{t-h_{\Omega }}^{\Omega }$ and $Y_{t}^{\Omega }$. Consider the following two matrices:

(11) $$\begin{array}{cc} \Theta_{1}\left(\Sigma_{Z}^{\Omega },h\right)= & \;{\left\{\Sigma_{XX}^{\Omega }\left(0\right)\right\}}^{-\frac{1}{2}}\Sigma_{XY}^{\Omega }\left(h\right){\left\{\Sigma_{YY}^{\Omega }\left(0\right)\right\}}^{-1}\Sigma_{YX}^{\Omega }\left(h\right){\left\{\Sigma_{XX}^{\Omega }\left(0\right)\right\}}^{-\frac{1}{2}},\;\mathrm{and}\;\\ \Theta_{2}\left(\Sigma_{Z}^{\Omega },h\right)= & \;{\left\{\Sigma_{YY}^{\Omega }\left(0\right)\right\}}^{-\frac{1}{2}}\Sigma_{YX}^{\Omega }\left(h\right){\left\{\Sigma_{XX}^{\Omega }\left(0\right)\right\}}^{-1}\Sigma_{XY}^{\Omega }\left(h\right){\left\{\Sigma_{YY}^{\Omega }\left(0\right)\right\}}^{-\frac{1}{2}}. \end{array}$$

A simple calculation reveals that the solution to the maximization problem in (10) is given by the eigenvalue decomposition of the matrices $\Theta_{1}\left(\Sigma_{Z}^{\Omega },h\right)$ and $\Theta_{2}\left(\Sigma_{Z}^{\Omega },h\right)$. In fact, the CBC ϕ(Ω) is the largest eigenvalue of $\Theta_{1}\left(\Sigma_{Z}^{\Omega },h\right)$ and the canonical directions $u_{\Omega }$ and $v_{\Omega }$ are given by the leading eigenvectors of $\Theta_{1}\left(\Sigma_{Z}^{\Omega },h\right)$ and $\Theta_{2}\left(\Sigma_{Z}^{\Omega },h\right)$, respectively (see [22]).

### 3.3. KenCoh: A Robust Estimator of Canonical Band Coherence

Now, we present a robust estimation method of the CBC. First, note that an estimate of the CBC $\hat{\phi }\left(\Omega \right)$ is obtained by considering the eigenvalue decomposition of the matrices $\Theta_{1}\left({\hat{\Sigma }}_{Z}^{\Omega },h\right)$ and $\Theta_{1}\left({\hat{\Sigma }}_{Z}^{\Omega },h\right)$, where ${\hat{\Sigma }}_{Z}^{\Omega }\left(h\right)$ is estimated from the observed data. In a classical approach, the sample variance–covariance matrix is used to estimate the above matrices. However, the estimator is sensitive to the heavy-tailed properties of the stochastic process and breaks down in the presence of outliers. To circumvent this problem, robust estimators for the covariance matrix can be used, e.g., the minimum covariance determinant estimator [23] and minimum volume ellipsoid estimator [24]. In this article, we present a robust estimator defined in [22] that is based on Kendall’s τ rank correlation coefficient for time series data (also see [25,26]).

We assume that the distribution of $Z_{t}^{\Omega }$ is elliptically symmetric with density generator $\psi :R_{+}\to R_{+}$, location vector $\mu_{Z}^{\Omega }\in R^{d}$, and the d×d positive definite scale matrix $\Lambda_{Z}^{\Omega }$ for all t≥1 [27]. It follows from the properties of an elliptically symmetric distribution that $\Sigma_{Z}^{\Omega }\left(h\right)=-2\psi^{\prime }\left(0\right)\Lambda_{Z}^{\Omega }\left(h\right)$ where $\Lambda_{Z}^{\Omega }\left(h\right)$ is the cross-scale matrix between $Z_{t-h}^{\Omega }$ and $Z_{t}^{\Omega }$, and $\psi^{\prime }\left(0\right)$ is the first derivative of the density generator evaluated at zero. We consider $\Theta_{i}\left(\Lambda_{Z}^{\Omega },h\right)$ similar to $\Theta_{i}\left(\Sigma_{Z}^{\Omega },h\right)$, i=1,2 as defined in Equation (11). Observe that

$$\begin{array}{c} \Theta_{1}\left(\Sigma_{Z}^{\Omega },h\right)=\Theta_{1}\left(\Lambda_{Z}^{\Omega },h\right),\;\mathrm{and}\;\Theta_{2}\left(\Sigma_{Z}^{\Omega },h\right)=\Theta_{2}\left(\Lambda_{Z}^{\Omega },h\right). \end{array}$$

We further assume that the diagonal elements of the matrix $\Lambda_{Z}^{\Omega }\left(0\right)$ are all 1, and the off-diagonals of $\Lambda_{Z}^{\Omega }\left(h\right)$ all lie strictly between −1 and 1 for all h∈Z. Motivated by Theorem 3.1 of [27], we estimate the j,k-th element of the d×d matrix $\Lambda_{Z}^{\Omega }\left(h\right)$ as the following:

(12) λ^jkΩ(h)=sinπ2τ^jkΩ(h),whereτ^jkΩ(h)=1T2∑1≤t<s≤Tsign{(Zj,t−hΩ−Zj,s−hΩ)(Zk,tΩ−Zk,sΩ)}.

Here, the random variable ${\hat{\tau }}_{jk}^{\Omega }\left(h\right)$ captures the monotone association between $Z_{j,t-h}^{\Omega }$ and $Z_{k,t}^{\Omega }$. Since it is based on the signs of the observed signals, it is robust to outliers present in the data. As a result, the estimator ${\hat{\Lambda }}_{Z}^{\Omega }\left(h\right)={\hat{\lambda }}_{jk}^{\Omega }\left(h\right)$ and subsequently, ${\hat{\Theta }}_{1}\left(\Lambda_{Z}^{\Omega },h\right)$ and ${\hat{\Theta }}_{2}\left(\Lambda_{Z}^{\Omega },h\right)$ are also free from the influence of outliers. Thus, the estimation method leads to a robust analysis of the canonical band coherence. Since the estimator is based on Kendall’s τ, we refer to this method as KenCoh. The performance of KenCoh is discussed in the following subsection.

Several robust alternatives to the proposed estimator of CBC can be considered in practice. A straightforward approach is to use a robust estimator for the covariance matrix $\Sigma_{Z}^{\Omega }\left(h\right)$, such as the minimum covariance determinant (MCD) estimator proposed by [24]. However, MCD has a high computational cost, growing polynomially as $O(T^{\nu })$, where ν=d(d+3)/2 [28]. Another approach to robustifying canonical coherence analysis is to replace Pearson’s correlation with a more robust measure of association, such as Spearman’s rank correlation between $u^{⊤}X_{t-h}^{\Omega }$ and $v^{⊤}Y_{t}^{\Omega }$. While finding the optimal canonical directions remains challenging in practice, a reasonable approximation can be achieved by restricting the search space to a finite set. However, estimating Spearman’s correlation has a computational complexity of $O(T^{3}\log T)$, which becomes impractical for large samples. In contrast, KenCoh has a significantly lower complexity of O(TlogT), making it a more scalable option for large datasets.

### 3.4. Application to LFP Data

We apply the robust methodology of Section 3.3 to the LFP data collected from Buchanan for the 270 trials. Buchanan was able to identify 232 of them correctly (203 in-sequence trials and 29 out-of-sequence trials). On average, he has a higher rate of responding correctly when the odors were presented in-sequence (i.e., 90% success rate) than when odors were not in-sequence (i.e., 66% success rate). Our goal is to study the spectral association between proximal and distal regions (see Figure 6) and characterize the connectivity structure of the tetrodes in respective regions for in-sequence and out-of-sequence trials. This involves estimating the canonical directions for five odors and two types of trials.

#### 3.4.1. Test of Hypotheses

We consider the beta band (Ω=(12–30) Hz) for the real data analysis. Recall that the beta band is known to dominate the signal during task and concentration [29]. Let $u_{\Omega }^{\left(g,s\right)}={\left(u_{1,\Omega }^{\left(g,s\right)},\ldots ,u_{11,\Omega }^{\left(g,s\right)}\right)}^{⊤}$ be the true canonical direction associated with the proximal region for odor *g*, and *s* type of trial, where g∈{A,B,C,D,E}, and s={I,O}, such that, *I* stands for in-sequence and *O* for out-of-sequence trials. Similarly, let $v_{\Omega }^{\left(g,s\right)}={\left(v_{1,\Omega }^{\left(g,s\right)},\ldots ,v_{9,\Omega }^{\left(g,s\right)}\right)}^{⊤}$ denote the true canonical direction associated with the distal region. We are particularly interested in the configuration of different channels that lead to maximum band coherence. To identify which channels’ contribution differ across different odors and types of trials, we conduct tests for the hypotheses

(13) $$\begin{array}{c} H_{0,s}^{g,k}:\left\{u_{\Omega }^{\left(g,s\right)},v_{\Omega }^{\left(g,s\right)}\right\}=\left\{u_{\Omega }^{\left(k,s\right)},v_{\Omega }^{\left(k,s\right)}\right\}\;\;\mathrm{vs}\;\;H_{1,s}^{g,k}:\left\{u_{\Omega }^{\left(g,s\right)},v_{\Omega }^{\left(g,s\right)}\right\}\neq \left\{u_{\Omega }^{\left(k,s\right)},v_{\Omega }^{\left(k,s\right)}\right\} \end{array}$$

for all g≠k∈{A,B,C,D,E} and s∈{I,O}.

Let ${\left\{X_{t}^{\left(r\right)}\right\}}_{t=1}^{T}$ and ${\left\{Y_{t}^{\left(r\right)}\right\}}_{t=1}^{T}$ denote the observed signals during the *r*-th trial corresponding to proximal and distal tetrodes, respectively with T=1000 for all r≥1. Let ${\hat{u}}_{\Omega }^{\left(g,s\right)}\left(r\right)$ and ${\hat{v}}_{\Omega }^{\left(g,s\right)}\left(r\right)$ be the estimated canonical directions associated with the proximal and distal regions, respectively, for trial *r*, odor *g*, and *s* type of trial, where $r=1,\ldots ,R_{g}^{\left(s\right)}$. Here, we assume that the trials are independent and identical. Therefore, ${\hat{u}}_{\Omega }^{\left(g,s\right)}\left(r\right)$ and ${\hat{v}}_{\Omega }^{\left(g,s\right)}\left(r\right)$ are independently and identically distributed random vectors for all $r=1,\ldots ,R_{g}^{\left(s\right)}$. We further consider the multivariate spatial median [30] based on the random samples $\left\{u_{\Omega }^{\left(g,s\right)}\left(r\right):r=1,\ldots ,R_{g}^{\left(s\right)}\right\}$, $\left\{v_{\Omega }^{\left(g,s\right)}\left(r\right):r=1,\ldots ,R_{g}^{\left(s\right)}\right\}$ as robust estimators of the canonical directions $u_{\Omega }^{\left(g,s\right)}\left(r\right)$ and $v_{\Omega }^{\left(g,s\right)}\left(r\right)$. We define

$$\begin{array}{c} {\hat{U}}_{\Omega }^{\left(g,s\right)}=\underset{\mu \in R_{+}^{P}}{arg min}\sum_{r=1}^{R_{g}^{\left(s\right)}}∥{\hat{u}}_{\Omega }^{\left(g,s\right)}{\left(r\right)-\mu ∥}_{L_{1}}\;\;\mathrm{and}\;\;{\hat{V}}_{\Omega }^{\left(g,s\right)}=\underset{\mu \in R_{+}^{Q}}{arg min}\sum_{r=1}^{R_{g}^{\left(s\right)}}{∥{\hat{v}}_{\Omega }^{\left(g,s\right)}\left(r\right)-\mu ∥}_{L_{1}}. \end{array}$$

Finally, the test-statistic $T_{\Omega }^{\left(g,k,s\right)}$ is defined as

(14) $$T_{\Omega }^{\left(g,k,s\right)}=\frac{∥{\hat{U}}_{\Omega }^{\left(g,s\right)}-{\hat{U}}_{\Omega }^{\left(k,s\right)}{∥}_{L_{2}}}{\sqrt{P}}+\frac{∥{\hat{V}}_{\Omega }^{\left(g,s\right)}-{\hat{V}}_{\Omega }^{\left(k,s\right)}{∥}_{L_{2}}}{\sqrt{Q}},$$

for g≠k∈{A,B,C,D,E} and s={I,O}. The null hypothesis $H_{0,s}^{g,k}$ is rejected if the observed $T_{\Omega }^{\left(g,k,s\right)}$ is large. The cut-off is obtained by the permutation test where the labels *g* and *k* are randomly permuted to derive the distribution of $T_{\Omega }^{\left(g,k,s\right)}$ under the null hypothesis. The *p*-values in this multiple hypotheses testing framework are adjusted using the Benjamini–Hochberg method [31]. We apply the Synthetic Minority Over-sampling Technique (SMOTE), introduced by Chawla et al. [32], to address data imbalance for pairs of odors *g* and *k* with highly imbalanced sample sizes.

#### 3.4.2. Discussion

**Figure 7:**
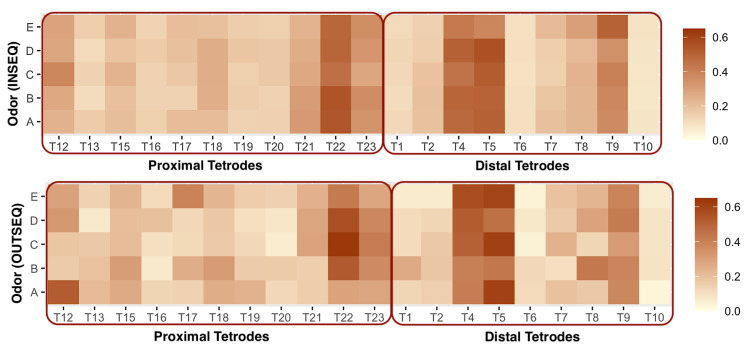
Multivariate spatial median of absolute canonical directions in the 12–30 Hz (beta band) frequency range for in-sequence (**top**) and out-of-sequence (**bottom**) odor presentation, for odors A–E.

**Figure 8:**
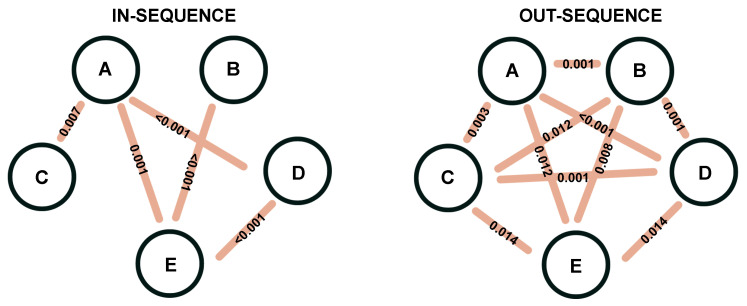
Significant adjusted *p*-values obtained using the permutation test for the five odors presented in-sequence (**left panel**) and out-of-sequence (**right panel**).

In Figure 7, we draw a heatmap of the estimates $\left\{|{\hat{U}}_{\Omega }^{\left(g,s\right)}|,|{\hat{V}}_{\Omega }^{\left(g,s\right)}|\right\}$ for s∈{I,O}, and g∈{A,B,C,D,E}. The top panel shows subtle changes in brain connectivity when odors are presented in sequence. In contrast, brain connectivity shows more pronounced changes for different odors when presented out of sequence (bottom panel). For in-sequence, we also observe that common tetrodes drive the connectivity between proximal and distal regions for all odors, e.g., T22, T4, and T5. On the other hand, for out-of-sequence trials, the relative contribution of tetrodes varies across odors. For instance, the contribution of T12 and T17 to odors A and E, respectively, is substantially higher than their contribution to the other odors (see bottom panel, Figure 7). The significance of the changes in the contribution is tested using the permutation test described in Section 3.4.1. The results of the tests are summarized in Figure 8.

We perform pairwise comparisons between odors separately for in-sequence and out-of-sequence trials. Figure 8 provides a graphical representation of our findings. The five odors are depicted as nodes, with edges connecting pairs of odors whose associated brain connectivity structures differ significantly. For instance, the brain connectivity structures associated with odors A and B are not significantly different during in-sequence trials, which explains the absence of an edge between them. However, these structures differ significantly when the odors are presented out of sequence. In contrast, we observe a significant difference in the connectivity structures for odors A and C in both in-sequence and out-of-sequence trials.

Figure 8 reveals that, for in-sequence trials, the odors presented first and last (A and E) play a significant role, as their associated brain connectivity structures show notable differences. In contrast, no significant changes in brain connectivity structures are observed for odors presented in the middle of the in-sequence trials. However, the pattern shifts in out-of-sequence trials, where all odors exhibit distinct brain connectivity structures.

## 4. Granger Causality Across Node/Region Subsets

Recent brain connectivity analyses frequently involve high-dimensional signals, such as large sets of LFPs or EEG recordings. Dissecting the directional influence between specific nodes (i.e., channels, electrodes, tetrodes) or sub-regions (e.g., pre-defined cluster of nodes) within such high-dimensional and possibly complex networks can serve as a methodological basis for explaining the neural mechanisms at a detailed level. However, analyzing a pair of nodes or sub-regions under the possible confounding effects of other channels in the network leads to inferential complications. Various methods have addressed similar problems in high-dimensional networks [33,34,35,36,37,38], yet the difficulty of isolating a subset of nodes from the rest of the network remains a critical problem [39,40].

This section introduces an approach designed to overcome these challenges using spectral domain dynamic principal component analysis (sDPCA) [21,41,42]. The methodology aims to isolate two nodes (or sub-regions, depending on the context) of interest within a high-dimensional network by removing the aggregate influence of all other nodes, facilitating the subsequent application of conventional examinations for Granger causality (GC). By collapsing the complexity of the entire network into a low-dimensional representation and partialling out the effects of other nodes/subregions, the resulting node-specific signals are isolated from the confounding effects. This provides a practical medium for inferring directional interactions.

### 4.1. Inference in High-Dimensional Setting

Consider a high-dimensional network, G, of *P* nodes (i.e., channels, electrodes, and tetrodes), each corresponding to a signal. Let the network, G, be $\left\{X_{p,t},X_{q,t},\zeta_{1,t},\ldots ,\zeta_{P-2,t}\right\}$, where $X_{p}=\left\{X_{p,t}\right\}$ and $X_{q}=\left\{X_{q,t}\right\}$ are two nodes of interest (NOIs) and $\zeta_{1,t},\ldots ,\zeta_{P-2,t}$ represent a large set of other nodes whose influence we wish to control.

We propose combining GC with sDPCA specifically to focus on pairwise interactions among NOI in LFP data. In general, LFP or brain imaging data often involve many channels/nodes measuring neural activity across multiple frequency bands, making direct pairwise GC analysis difficult because of the curse of dimensionality and the risk of overfitting or spurious connections. Applying sDPCA provides an advertent dimensionality reduction step with respect to the spectral structure of the data: sDPCA operates in the frequency domain and accounts for the dominant oscillatory and frequency-specific patterns in the signals. This means that important neural dynamics (such as rhythmic oscillations or cross-frequency interactions) are preserved in a few components rather than averaged out. Following the implementation of sDPCA to extract and regress the network’s interfering background from each node of interest, the exploration of GC between the residual signals becomes feasible. While alternative approaches exist (for example, one could apply GC with sparse regularization to the complete set of channels), the sDPCA+GC methodology is proposed for its ability to maintain frequency-specific information and improve reliability in detecting neural interactions. This balanced and easy-to-implement strategy addresses the complexity of LFP data by focusing on physiologically meaningful components, thus providing a clearer and more interpretable pairwise causal connectivity analysis in a high-dimensional neural recording context.

To isolate the NOI and uncover their causal relationships, one can follow a strategy that involves transforming the signals as follows:

$$\begin{array}{cc} X_{p}^{*} & =X_{p}-E\left(X_{p}|encapsulate_{\zeta }\left(\zeta_{1,t},\ldots ,\zeta_{P-2,t}\right)\right),\\ X_{q}^{*} & =X_{q}-E\left(X_{q}|encapsulate_{\zeta }\left(\zeta_{1,t},\ldots ,\zeta_{P-2,t}\right)\right), \end{array}$$

where $encapsulate_{\zeta }\left(\cdot \right)$ is a function summarizing the collective influence of the remaining nodes excluded. After this step, $X_{p}^{*}$ and $X_{q}^{*}$ become isolated versions of the original signals, where the network’s background variability has been partially removed. The key question is how to construct this function $encapsulate_{\zeta }\left(\cdot \right)$ to capture the large network’s dynamics without overfitting or losing crucial frequency-dependent structures.

### 4.2. Using Spectral Dynamic PCA to Represent the Background Network

Conventional principal component analysis (PCA) focuses on reducing dimensionality by finding linear combinations of variables that explain the most significant variance. However, PCA operates on covariance structures that do not directly incorporate temporal dependency or frequency-specific patterns. Neural signals often have rich spectral content—specific frequencies may carry more meaningful interactions than others. Frequency-domain dynamic PCA (sDPCA) [21,42,43] addresses this need by operating in the frequency domain and extracting components that are informative about temporal associations.

To apply sDPCA, we first estimate the cross-spectral density matrix of the background signals $\zeta_{t}={\left(\zeta_{1,t},\ldots ,\zeta_{P-2,t}\right)}^{⊤}$. Let $S_{\zeta }\left(\omega \right)$ be the cross-spectral density matrix at frequency ω. This matrix encodes frequency-specific variances and covariances. It can be estimated by

$${\hat{S}}_{\zeta }\left(\omega \right)=\sum_{|h|\leq M}w\left(\frac{\left|h\right|}{M}\right){\hat{\Sigma }}_{\zeta }\left(h\right)\exp\left(-2\pi ih\omega \right),$$

where w(·) is a window function, *M* is the window size, and ${\hat{\Sigma }}_{\zeta }\left(h\right)$ is the empirical lag-*h* covariance matrix given by

$${\hat{\Sigma }}_{\zeta }\left(h\right)=\frac{1}{T}\sum_{t=1}^{T-|h|}\left(\zeta_{t+|h|}-\bar{\zeta }\right){\left(\zeta_{t}-\bar{\zeta }\right)}^{⊤},$$

for h≥0, and

$${\hat{\Sigma }}_{\zeta }\left(h\right)={\hat{\Sigma }}_{\zeta }{\left(-h\right)}^{⊤}=\frac{1}{T}\sum_{t=1}^{T+h}\left(\zeta_{t}-\bar{\zeta }\right){\left(\zeta_{t-h}-\bar{\zeta }\right)}^{⊤}$$

for h<0.

At each frequency ω, we solve the eigenvalue problem:

$$S_{\zeta }\left(\omega \right)\varphi_{j}\left(\omega \right)=\lambda_{j}\left(\omega \right)\varphi_{j}\left(\omega \right).$$

The eigenvectors $\varphi_{j}\left(\omega \right)$ represent frequency-domain principal directions, and $\lambda_{j}\left(\omega \right)$ are the corresponding eigenvalues. These frequency-specific eigenvectors reflect how variability is arranged throughout the spectral domain.

To return to the time domain, we compute filters from the eigenvectors via inverse Fourier transform:

$$\varrho_{m}^{\left(j\right)}=\frac{1}{2\pi }\int_{-\pi }^{\pi }\varphi_{j}\left(\omega \right)e^{-im\omega }d\omega ,$$

for integer shifts *m*. Each set of filters,

$$\{\varrho_{m}^{\left(j\right)}:m=-L,\ldots ,L\},$$

defines a dynamic principal component in the time domain.

Applying these filters to $\zeta_{t}$ yields a reduced set of dynamic principal component scores:

$${\mathrm{dpc}}_{j,t}=\sum_{m=-L}^{L}{\left\{\varrho_{m}^{\left(j\right)}\right\}}^{⊤}\zeta_{t-m}$$

Only a few dynamic components are usually necessary to capture a significant fraction of the total variance. These scores represent a low-dimensional snapshot of the entire background network’s activity, integrated over time and frequency.

### 4.3. Partialling Out Background Influence and Applying Granger Causality

Once we have obtained principal scores $\left\{{\mathrm{dpc}}_{1,t},\ldots ,{\mathrm{dpc}}_{sc,t}\right\}$, where sc≤P−2, treated as covariates summarizing the background nodes, we model

$$E\left(X_{p}|{\mathrm{dpc}}_{1,t},\ldots ,{\mathrm{dpc}}_{sc,t}\right)\;\mathrm{and}\;E\left(X_{q}|{\mathrm{dpc}}_{1,t},\ldots ,{\mathrm{dpc}}_{sc,t}\right).$$

Subtracting these fitted values from $X_{p}$ and $X_{q}$ yields $X_{p}^{*}$ and $X_{q}^{*}$, which are now approximately isolated from the rest of the network’s influence. In practice, these conditional expectations are approximated by regressing each node of interest (e.g., $X_{p}$) on the set of dynamic principal component scores $\left\{{\mathrm{dpc}}_{1,t},\ldots ,{\mathrm{dpc}}_{sc,t}\right\}$ and their interactions. Using a linear model (or a nonlinear model by preference), the fitted values ${\hat{X}}_{p}$ and ${\hat{X}}_{q}$ are used to approximate $E(X_{p}|{\mathrm{dpc}}_{1,t},\ldots ,{\mathrm{dpc}}_{sc,t})$ and $E(X_{p}|{\mathrm{dpc}}_{1,t},\ldots ,{\mathrm{dpc}}_{sc,t})$, respectively.

With $X_{p}^{*}$ and $X_{q}^{*}$ in hand, we return to a more conventional Granger causality framework [44]. Testing for GC typically consists of comparing a restricted model that predicts $X_{p}^{*}$ using only its own past against an unrestricted model that also includes $X_{q}^{*}$’s past values:

$$\begin{array}{cc} \mathrm{Restricted}\text{:}\; & X_{p,t}^{*}=\gamma_{0}+\sum_{i=1}^{d_{X_{p}^{*}}}\gamma_{i}X_{p,(t-i)}^{*}+\eta_{t},\\ \mathrm{Unrestricted}\text{:}\; & X_{p,t}^{*}=\alpha_{0}+\sum_{i=1}^{d_{X_{p}^{*}}}\alpha_{i}X_{p,(t-i)}^{*}+\sum_{j=1}^{d_{X_{q}^{*}}}\beta_{j}X_{q,(t-j)}^{*}+\epsilon_{t}. \end{array}$$

If adding past values of $X_{q}^{*}$ significantly reduces variability in the predictive errors, we conclude that $X_{q}^{*}$ Granger causes $X_{p}^{*}$. Similarly, we can test whether $X_{p}^{*}$ Granger causes $X_{q}^{*}$.

**Figure 9:**
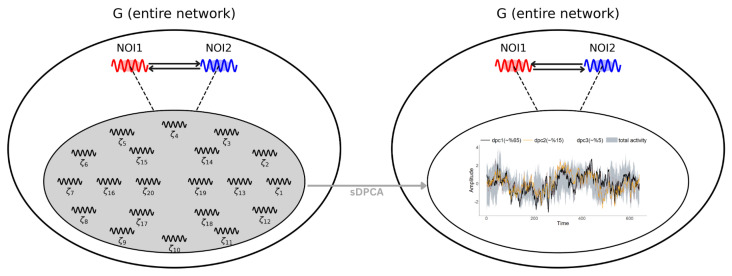
Granger causality in a high-dimensional network using the sDPCA approach.

Working with the isolated channels, $X_{p}^{*}$ and $X_{q}^{*}$, makes the GC results less likely to be distorted by unmodeled interactions from other network nodes. Thus, this approach transforms a high-dimensional problem into a more tractable one, employing the sDPCA spectrum-aware dimension reduction. Figure 9 exemplifies the proposed approach for GC in high-dimensional networks.

### 4.4. Practical Advantages and Limitations

The proposed methodology avoids the complexity of simultaneously fitting a high-dimensional vector autoregressive (VAR) model to the entire network. Instead, it establishes a controlled setting where the causal interactions between selected nodes can be tested more directly rather than being potentially affected by the influence of the entire network. It does so via bridging the original high-dimensional network and a low-dimensional summary captured by dynamic principal components. By partialling out these low-dimensional summaries, the nodes of interest recover with reduced confounding.

Thus, the sDPCA-based procedure is well suited for scenarios where interest focuses on the directional interaction between a small subset of channels embedded in an extensive network. It can also be adapted when the nodes of interest represent not just single channels but clusters of channels (i.e., regions), each represented by their sDPCA-derived summary scores.

However, several limitations could be noted. First, selecting an appropriate number of principal components (here denoted sc≤P−2) is critical; poor choices can lead to the omission of essential dynamics or the exclusion/inclusion of informative/uninformative components. Second, spectral estimation relies on smoothing parameters and windowing techniques, which may discard sharp spectral features or hidden localized frequency-specific phenomena. Third, conventional tests for GC assume linearity and stationarity, yet neural signals often violate these assumptions, thereby potentially invalidating the inferred causal connections. Finally, while sDPCA reduces the dimensionality in a frequency-aware mode, interpreting the resulting dynamic principal components and linking them to specific bio-physiological processes can remain a significant challenge.

### 4.5. Granular Level GC in Olfactory LFP Network

**Figure 10:**
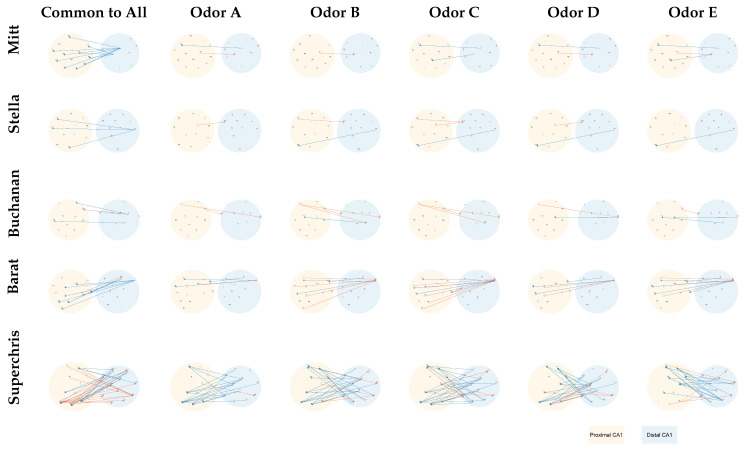
GC connectivities across odors A, B, C, D, and E for each subject.

We considered trials where the rats correctly identified a given odor’s sequence status (In-sequence—Correct, Table 1). For each trial of interest, segments of LFP signals are extracted starting at the onset of the odor. These segments are then differenced to ensure stationarity and combined across odor types and trials, ultimately yielding a separate collection of LFP samples for each odor. Because each subject’s recording involved multiple tetrodes placed along the proximal to distal axis of CA1, the goal is set to examine how specific tetrode pairs might exhibit directional influences/connectivities (i.e., Granger causality) unique to a particular odor or common across all odors. In practice, we conduct GC analysis for every distal–proximal (i.e., proximal–distal) pair and count how frequently one tetrode “drove” another across the trials for that odor. If a directed association is significant on at least 99% of trials, we label that connection as consistently present. This procedure “votes” on each possible directed relation in the network and flags only those consistently emerging in every trial. Finally, we compile these odor-specific GC connections into plots. The results are visualized as in Figure 10: each row corresponds to one subject, each column to odor, plus the first column shows a connectivity pattern recurring under all odor conditions.

Figure 10 reveals that each subject has certain distal (i.e., blue-colored region) to proximal (i.e., orange-colored region) channels linking up under every odor. Mitt’s distal tetrodes T1,T2 consistently influence proximal channels T12,…,T22. These connections emerge throughout odors A–E, whereas T1→T13 or T2→T23 occur exclusively in certain odors (e.g., odor A, C, E), implying that these unique routes are distinct circuit elements engaged by particular olfaction. Stella features T2→T20, T2→T21, and T2→T23 across all odors, with distinct connectivities such as T17→T10 or T2→T22 emerging in certain odor, suggesting that while Stella’s distal to proximal drive persists, some odor-specific flows may support specific olfaction demands. Buchanan displays a smaller cluster of common edges than Mitt or Stella, but we still see consistent patterns like T1→T16 or T2→T21 or T21→T6. Odor-specific differences appear in connections such as T12→T4 for odor A/C/D, T1→T13 for odor B/E, and T4→T23 for odor D/E. While these odor-level connections vary, the overall structure still centers on distal nodes (T1, T2, T4), influencing its several proximal tetrodes T16, T21, T22, and T6. Barat has relatively a large cluster of distal-to-proximal associations, with T1 consistently targeting T12,…,T19, and T2 targeting T20,T21,T22,T23. These connections remain steady across odors. A few bidirectional connections (e.g., T19↔T1 and T22↔T2) also appear. Some extra pathways show up in certain odors (e.g., T20→T1 in odor C, T21→T4 in odor C, and T19→T1 in odor A/E), but the broader pattern remains T1,T2 to T12,…,T22. The subject Superchris stands apart by exhibiting a large cluster of bidirectional connectivity between its distal tetrodes (i.e., T12,…,T23) and proximal tetrodes (i.e., T1, T2, …, T10). Unlike Mitt or Stella, who rely more on unidirectional distal → proximal pathways (for example, T1→T12 in Mitt or T2→T20 in Stella), Superchris shows multiple two-way interactions, such as T1↔T14 and T2↔T21, that persist under all odors. Superchris also exhibits several odor-specific connections involving T6, T7, T8, or T9, which emerge only in certain odors, whereas other subjects generally employ fewer links when shifting from one odor to another. Therefore, while the common pattern of distal → proximal influences remains visible, Superchris integrates richer reciprocal activity, suggesting a denser or interconnected CA1 network compared to the predominantly one-directional flows seen in Mitt, Stella, Buchanan, or Barat.

A crucial question here is whether odor-specific connections show a clear shift in CA1 circuitry. Considering Figure 10, most subjects display considerable similarities across “common to all odors” patterns (i.e., distal to proximal). This overlap suggests that hippocampal olfaction processing relies on shared associations of distal to proximal transmissions, with modest variations in direction or presence depending on odor identity. The new connections that do appear exclusively in a single odor could serve a more specialized role.

Consequently, a basic understanding is that each subject’s dorsal CA1 circuit favors a core route of information flow, with distinctive odor-related changes superimposed. In all five subjects, sub-regions along distal CA1 often serve as a “source” area projecting into multiple proximal tetrodes, while back-influences from proximal to distal also emerge in the data. This observation aligns with existing knowledge that CA1 exhibits prominent directionality along its anatomical axis [45,46].

## 5. Spectral Transfer Entropy

Unlike GC, which relies on model-based assumptions, transfer entropy (TE) is an information-theoretic measure that captures directional and potentially nonlinear dependencies between signals. This makes it particularly valuable for analyzing complex interactions in neural systems.

Consider two signals, denoted by $\left\{X_{q,t}\right\}$ and $\left\{X_{p,t}\right\}$, observed from distinct nodes, voxels, channels, or tetrodes in a brain network. Let

$$X_{q,t-k}={\left(X_{q,t-1},\ldots ,X_{q,t-k}\right)}^{⊤}\;\mathrm{and}\;X_{p,t-\ell }={\left(X_{p,t-1},\ldots ,X_{p,t-\ell }\right)}^{⊤},$$

for some time lags *k* and *ℓ*. Developed by Schreiber [47], TE quantifies the information transfer from $\left\{X_{q,t}\right\}$ to $\left\{X_{p,t}\right\}$ by measuring the conditional mutual information (CMI) between $X_{p,t}$ and $X_{q,t-k}$ given $X_{p,t-\ell }$. This is expressed as

$$TE\left(X_{q}\to X_{p}\;;\;k,\ell \right)=I\left(X_{p,t};X_{q,t-k}|X_{p,t-\ell }\right),$$

where I(·;·∣·) represents the conditional mutual information. This metric reflects the directed influence of $X_{q}$ on $X_{p}$ while accounting for the past states of $X_{p}$. More precisely,

$$\begin{array}{cc} I(X_{p,t},X_{q,t-k}|X_{p,t-\ell }) & =\;\int \int \int \;f\left(x_{p},{x^{\prime }}_{p},{x^{\prime }}_{q}\right)\log\frac{f\left({x^{\prime }}_{p}\right)f\left(x_{p},{x^{\prime }}_{p},{x^{\prime }}_{q}\right)}{f\left(x_{p},{x^{\prime }}_{p}\right)f\left({x^{\prime }}_{p},{x^{\prime }}_{q}\right)}\;dx_{p}d{x^{\prime }}_{q}d{x^{\prime }}_{p}, \end{array}$$

with $x_{p}=x_{p,t}$, ${x^{\prime }}_{p}={\left(x_{p,t-1},\ldots ,x_{p,t-\ell }\right)}^{⊤}$ and ${x^{\prime }}_{q}={\left(x_{q,t-1},\ldots ,x_{q,t-k}\right)}^{⊤}$. For a comprehensive discussion on other information-theoretic measures, including entropy and mutual information, refer to Cover and Thomas [48]. In contrast with GC, which looks at the improvement in the prediction variance due to the additional information provided by another series’ history, TE measures the causal impact of a series to another series directly from their joint and conditional distributions. Explicitly, TE quantifies the statistical conditional dependence of $X_{p,t}$ on the past $X_{q,t-k}$ given its own history $X_{p,t-\ell }$. This formulation does not require any assumption on the distribution (e.g., Gaussianity) or the type of relationship (e.g., linear) between the two series, hence making the TE framework more general and applicable to analyzing complex data like LFP signals.

When the interest lies in relating effective connectivity to various frequency bands with well-explored cognitive interpretations, one strategy is to apply a bandpass filter on the observed signals to extract the band-specific oscillations of interest and conduct investigations via the GC or TE framework. For example, one may consider the filtered series $X_{q,t}^{\Omega }$ and $X_{p,t}^{\Omega }$, where their respective spectral densities concentrate only on frequency band Ω, and calculate TE from $X_{q,t}^{\Omega }$ to $X_{p,t}^{\Omega }$. However, the smooth oscillatory behavior of these band-specific oscillations often leads to erroneous results for approaches like GC and TE as linear filtering induces potential temporal dependence distortion and the false extraction of spectral influence [49,50,51]. The problem does not stem from these causal frameworks (i.e., GC and TE) but rather from the direct use of smoothly oscillating filtered signals.

To address this issue, Redondo et al. [52] formulated the spectral transfer entropy (STE) measure. Instead of capturing the direction and magnitude of information flow directly between two band-specific series, STE defines the information transfer between two nodes of a brain network based on a series of maximum amplitudes over non-overlapping time blocks. Let $Y_{q,b}^{\Omega }=\max\left(|X_{q,t}^{\Omega }|;t\in \left\{t_{b}+1,\ldots ,t_{b}+m\right\}\right)$ and $Y_{p,b}^{\Omega }=\max(\left|X_{p,t}^{\Omega }\right|;$$t\in \{t_{b}+1,\ldots ,t_{b}+m\})$ where $t_{b}$ is the time point preceding the *b*-th time block of length *m*. Concisely, $\left\{Y_{q,b}^{\Omega }\right\}$ and $\left\{Y_{p,b}^{\Omega }\right\}$ represent the block maxima series of amplitudes of the oscillations $X_{q,t}^{\Omega }$ and $X_{p,t}^{\Omega }$, respectively. Specifically, STE from $X_{q,t}^{\Omega }$ to $X_{p,t}^{\Omega }$, denoted by $STE_{\Omega }\left(X_{q}\to X_{p}\;;\;k,\ell \right)$, is defined as

$$\begin{array}{c} STE_{\Omega }\left(X_{q}\to X_{p}\;;\;k,\ell \right)=TE\left(Y_{q}^{\Omega }\to Y_{p}^{\Omega }\;;\;k,\ell \right), \end{array}$$

and is shown to be robust (empirically) to the inherent issues associated with linear filtering, i.e., it adequately captures spectral causal influences with controlled false positive rates, which provides evidence of the practical advantages of such a formulation.

**Figure 11:**
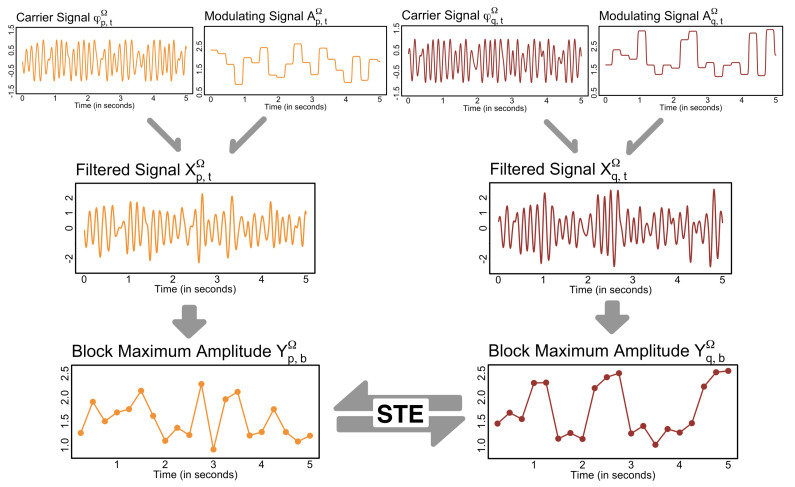
Illustration of carrier signals and modulating signals producing the band-specific oscillations and the aggregation to series of maximum amplitude over non-overlapping time blocks for the STE measure.

Aggregating band-specific signals into a series of maximum amplitudes over time blocks takes inspiration from communication theory, where the information transfer between two devices occurs through signal modulation. That is, an Ω-band oscillation $X_{t}^{\Omega }$ can be expressed as a product of a carrier signal $\varphi_{t}^{\Omega }$, whose spectral density is concentrated in the frequency band Ω and serves as the information pathway for the flow of information, and a modulating signal $A_{t}^{\Omega }$ that carry the information being transferred from one node to another (see Figure 11 for illustration). However, there is a shift in the temporal resolution of causality defined by the STE measure. For instance, if the signals are observed at a sampling rate of 1000 Hz (i.e., at 1000 time points per second) and the specified block size is m=100, STE quantifies the causal interactions that occur in about every one-tenth of a second (which is slower than the original temporal scale of the observed data). For more details on the interpretation, choice of tuning parameters, and vine copula-based inference for STE, which we employ in the subsequent analysis, refer to [52].

Let $X_{q,t}$ and $X_{p,t}$ be the LFP signals from tetrode *q* in the distal region and tetrode *p* in the proximal region, respectively. In this section, our primary objective is to identify differences in effective connectivity between tetrodes placed in the distal and proximal regions during correct and incorrect trials of mice given the olfactory-based task. Correct trials refer to trials where a mouse received a reward for recognizing an in-sequence odor, while incorrect trials result in having no reward. Here, we consider in-sequence trials from two subjects (namely *Superchris* and *Mitt*) given the odors *rum* and *lemon*, respectively. Since the number of incorrect trials is much smaller than that of the correct trials, we randomly sample an equal number of correct trials to make balanced cases. To be exact, we include 9 trials each from Superchris and 13 trials each from Mitt in the analysis. However, each trial contains roughly 1.2 s of LFP recordings after the odor presentation. Since the STE framework requires aggregating data over non-overlapping time blocks of length m=100 (which we specify to define a practical temporal resolution of causality), we use all block maxima series obtained from all correct trials and from all incorrect trials to calculate the STE measure. Finally, we focus our attention on two frequency bands, namely the alpha and beta bands, as several spectral analyses on individual LFP signals reveal changes in the latter related to olfactory functions [53,54,55], while differences in the former are yet to be discovered. Our goal is to provide insights on the causal influence of these band-specific oscillations on one another via the distributions of $STE_{\alpha }\left(X_{q}\to X_{p}\right)$ and $STE_{\beta }\left(X_{q}\to X_{p}\right)$ across relevant {q,p}-tetrode pairs, complimenting the existing results based on univariate spectral methods.

**Figure 12:**
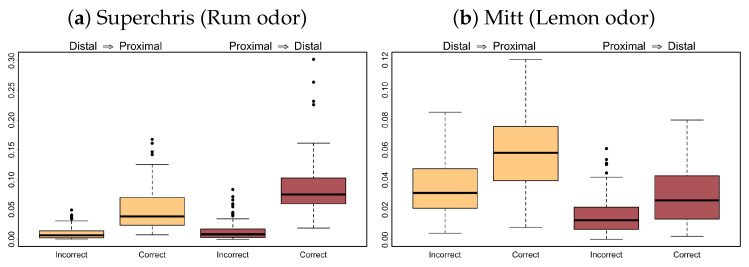
Distribution of $STE_{\alpha }\left(X_{q}\to X_{p}\right)$ (**alpha band**) across all relevant “distal–proximal”-tetrode pairs for correct and incorrect in-sequence trials of subjects (**a**) Superchris presented with the rum odor, and (**b**) Mitt presented with the lemon odor.

**Figure 13:**
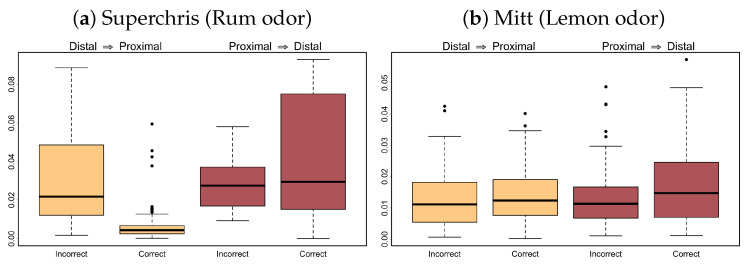
Distribution of $STE_{\beta }\left(X_{q}\to X_{p}\right)$ (**beta band**) across all relevant “distal–proximal”-tetrode pairs for correct and incorrect in-sequence trials of subjects (**a**) Superchris presented with the rum odor, and (**b**) Mitt presented with the lemon odor.

In Figure 12, we observe a high magnitude of information transfer, as quantified by the STE measure, between the distal and proximal regions during correct in-sequence trials while lower STE values during incorrect in-sequence trials. In addition, the differences in the magnitude of captured causal influence between correct and incorrect trials are highly prominent for subject Superchris while being less yet arguably still prominent for subject Mitt. This suggests that the STE approach is able to reveal prominent differences in connectivity patterns among the multivariate LFP signals in the alpha band, even though there is limited work on univariate spectral density methods that detect any differences in the same frequency band. By contrast, the flow of information in the beta band from the distal to the proximal region of Superchris has higher magnitudes during incorrect trials than during correct trials, while the STE values from proximal to distal region have larger variability among correct trials than among incorrect trials (see Figure 13). Further, there are very minimal differences in the beta band for subject Mitt. Such inconsistency in differences may be related to the odors presented to the respective subjects, as one odor may have a stronger or weaker impact on the subjects than the other. Nonetheless, the STE framework is a promising new tool for investigating effective brain connectivity, which we illustrate as successful in providing insights into how node interactions in the frequency domain may vary among different brain networks.

## 6. Wavelet Coherence Analysis

**Figure 14:**
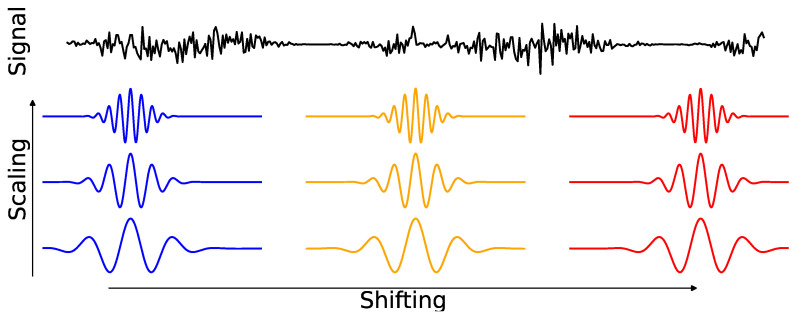
Non-stationary signal and its wavelet decomposition. The top panel displays a non-stationary signal, while the bottom panel shows multiple wavelet functions obtained by scaling and shifting a base wavelet. These wavelets act as time-localized filters that capture different features of the signal at various scales.

A key challenge in analyzing brain signals, such as LFPs, is their inherent non-stationarity; that is, statistical properties like the spectrum (or covariance) evolve over time. Wavelet analysis has proven exceptionally useful in capturing the transient features of non-stationary signals due to the compact support and flexibility of wavelet functions [56,57]. The compact support of wavelets allows for dynamic scaling—through compression or stretching as illustrated in Figure 14—which enables them to adapt to changing signal characteristics. In contrast, traditional Fourier methods, which lack time localization and the ability to adapt to a signal’s dynamic behavior, often struggle to capture these transient properties.

To address these limitations, Nason et al. [58] introduced a scale-specific stochastic representation of time series that leverages the multi-resolution property of wavelets to estimate evolving wavelet coherence. Building on this foundation, Park et al. [59] extended the framework to multivariate locally stationary wavelet (LSW) processes, enabling precise characterization of single-scale coherence among different channels. More recently, Wu et al. [60] proposed an innovative modeling framework that effectively captures the cross-scale dependence structure between channels in multivariate non-stationary time series. This advancement further enhances the capability of wavelet-based methods to uncover complex dependencies and evolving connectivity patterns in neural signals.

In this section, we introduce the framework of LSW and demonstrate its application to analyzing LFP data from different brain regions. We implement both single- and cross-scale coherence to capture the time-varying dependence structure across regions. This approach also allows us to examine how fluctuations in longer-term dynamics influence the amplitude of shorter-term dynamics, providing deeper insights into the multi-scale interactions within the brain.

### 6.1. LSW Model and Wavelet Coherence

Wavelets are powerful mathematical tools that enable the decomposition of signals into components containing both time and frequency (or scale) information. Unlike the Fourier transform, which represents signals as combinations of infinite sinusoids and provides only global frequency information, wavelets are uniquely suited for analyzing localized variations in signals. This makes wavelets particularly valuable for studying non-stationary data where signal characteristics may vary over time.

Wavelet analysis is built on two foundational functions: the *father wavelet* ϕ and the *mother wavelet* ψ. The father wavelet ϕ is designed to capture smooth, low-frequency components of a signal and integrate them into one, ensuring a focus on the overall trend. In contrast, the mother wavelet ψ integrates to zero and is responsible for extracting detailed, high-frequency components, thereby highlighting localized variations in the signal. To analyze signals at different resolutions, the mother wavelet is compressed and shifted to generate a family of *child wavelets*. These wavelets, indexed by a scale parameter *j* and a shift parameter *k*, are defined as

(15) $$\begin{array}{c} \psi_{j,k}\left(t\right)=2^{-j/2}\psi \left(\frac{t-2^{j}k}{2^{j}}\right),\;j=1,\ldots ,J \end{array}$$

where *J* denotes the maximum number of scales, and the scale *j* determines the resolution or level of detail captured by the wavelet, with smaller *j* values corresponding to coarser scales and larger *j* values corresponding to finer scales. The parameter *j* determines the translation of the wavelet in time, allowing for localized analysis across the signal.

Nason et al. [58] introduced the LSW framework, a novel representation for stochastic processes exhibiting complex, time-varying dynamics. Unlike traditional wavelet decomposition, which typically relies on decimated and orthogonal wavelet bases, the LSW framework employs non-decimated wavelet bases. This means that the wavelet bases in LSW are non-orthogonal across different scales and shifts, allowing for a more flexible representation of signals with intricate temporal structures. This unique feature makes LSW particularly well suited for analyzing non-stationary processes where traditional methods may fall short. Park et al. [59] extended the LSW to a multivariate setting to capture the time-varying scale-specific cross-dependence between the components from the signals among different channels. Here, we directly start by introducing this multivariate LSW (MvLSW).

The P-variate locally stationary wavelet process ${\left\{X\left(t\right)\right\}}_{t=1}^{T}$, defined by [58], where $T=2^{J},J\in \;N$ can be represented by,

$$X\left(t\right)=\sum_{j=1}^{\infty }\sum_{k}V_{j}\left(k/T\right)\psi_{j,t-k}z_{j,k},$$

where ${\left\{\psi_{j,t-k}\right\}}_{jk}$ is a set of discrete non-decimated wavelets; $V_{j}\left(k/T\right)$ is the time-dependent transfer function matrix. $z_{j,k}$ are uncorrelated random vectors with mean vector 0 and variance–covariance matrix equal to the P×P identity matrix. Furthermore, the scale-*j* subprocess of $X_{t;T}$ is defined as

$$\begin{array}{c} X_{j}\left(t\right)=\sum_{k}V_{j}\left(k/T\right)\psi_{j,t-k}z_{j,k}. \end{array}$$

The evolutionary spectrum matrix is defined based on the transfer function, which is used for quantifying the time-scale power of $X_{t;T}$. This local wavelet spectral (LWS) matrix is given by

$$\begin{array}{c} S_{j}\left(k/T\right)=V_{j}\left(k/T\right)V_{j}^{⊤}\left(k/T\right) \end{array}$$

where $V_{j}^{⊤}\left(k/T\right)$ denotes the transpose of $V_{j}\left(k/T\right)$. The random innovation term $z_{j,k}$ is assumed to be uncorrelated across different scales *j* and shifts *k*:

$$\begin{array}{c} \mathrm{Cov}\left(z_{j,k}^{\left(i\right)},z_{j^{\prime },k^{\prime }}^{\left(i^{\prime }\right)}\right)=\delta_{i,i^{\prime }}\delta_{j,j^{\prime }}\delta_{k,k^{\prime }}, \end{array}$$

where δ denotes the Kronecker delta function; however, one of the main limitations of this framework is its inability to capture cross-dependence between subprocesses at different scales, which can be an important measure of dependence.

To address this limitation, ref. [60] relaxed this assumption by defining the covariance matrix of $z_{j,k}$ using a general matrix $Q_{jj^{\prime }}\left(k/T\right)$. The dual-scale LWS matrix is then formulated as

$$\begin{array}{c} S_{jj^{\prime }}\left(k/T\right)=V_{j}\left(k/T\right)Q_{jj^{\prime }}\left(k/T\right)V_{j^{\prime }}^{⊤}\left(k/T\right). \end{array}$$

Based on the single- and cross-scale LWS matrix, the time-varying wavelet coherence is defined as

$$\begin{array}{cc} \rho_{j}\left(k/T\right) & =D_{j}\left(k/T\right)S_{j}\left(k/T\right)D_{j}\left(k/T\right),\;\mathrm{single}\text{-}\mathrm{scale}\\ \rho_{jj^{\prime }}\left(k/T\right) & =D_{j}\left(k/T\right)S_{jj^{\prime }}\left(k/T\right)D_{j^{\prime }}\left(k/T\right),\;\mathrm{cross}\text{-}\mathrm{scale} \end{array}$$

where the matrices $D_{j}\left(u\right)$ and $D_{j^{\prime }}\left(k/T\right)$ are diagonal with elements ${\left\{S_{j}^{\left(p,p\right)}\left(k/T\right)\right\}}^{\left(-1/2\right)}$ and ${\left\{S_{j^{\prime }}^{\left(q,q\right)}\left(k/T\right)\right\}}^{\left(-1/2\right)}$, respectively (see details in [60]). The wavelet coherence values range from −1 to 1, measuring the local single- or cross-scale dependence structure between channel *p* and channel *q* in multivariate time series. This framework offers a powerful way to study the time-varying connectivity between different brain regions, enabling insights into dynamic neural interactions. An empirical way to calculate localized measures of cross-scale dependence in the time domain is

(16) $$\begin{array}{c} \rho_{jj^{\prime }}^{\left(p,q\right)}\left(t/T\right)=|\frac{\mathrm{Cov}(X_{j}^{\left(p\right)}\left(t\right),X_{j^{\prime }}^{\left(q\right)}\left(t\right))}{\sqrt{\mathrm{Var}(X_{j}^{\left(p\right)}\left(t\right))}\sqrt{\mathrm{Var}(X_{j^{\prime }}^{\left(q\right)}\left(t\right))}}{|}^{2}. \end{array}$$

It is easy to see that if $j=j^{\prime }$, then $\rho_{jj^{\prime }}=\rho_{j}$, i.e., the cross-scale coherence becomes equal to the single-scale coherence.

### 6.2. Wavelet Coherence Analysis with LFP Data

In this part, we implement both single-scale and cross-scale wavelet coherence across different channels of the LFP data recorded from Superchris. The primary objective is to determine whether wavelet coherence can effectively reveal alterations in brain connectivity when the rat makes mistakes in odor sequence discrimination.

**Figure 15:**
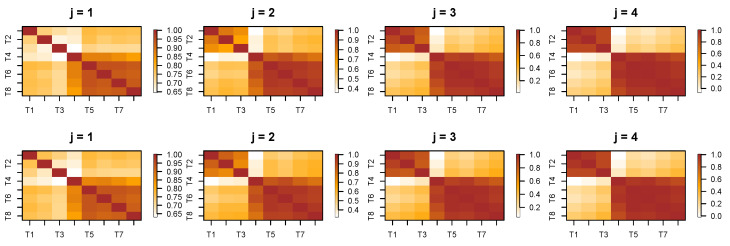
The single-scale coherence among the channels in LFP of Superchris; the first row is results based on trials with correct response in behavior test, and the second row corresponds to the wrong response.

**Figure 16:**
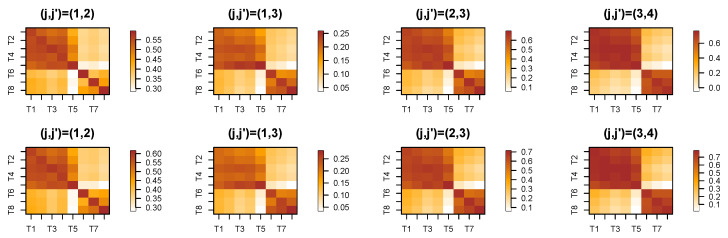
The cross-scale coherence among the given pair in LFP of Superchris corresponds to the trials that have a correct response and a wrong response, respectively.

The initial step involves decomposing the LFP time series into subprocesses at each scale. Here, we set the decomposition level to *J*, which can be adjusted based on the desired resolution of the analysis. Figure 15 presents the wavelet coherence between subprocesses at the same scale across eight channels recorded from Superchris, averaged over 24 trials of correct and incorrect responses, respectively. The heatmaps for the two groups of trials largely exhibit similar values across most blocks. Additionally, Figure 16 illustrates several pairs of wavelet coherence between subprocesses at different scales during trials where the rat responded correctly to the stimulus. The heatmap matrices are not symmetric as single-scale cases because of the changes in scales.

**Figure 17:**
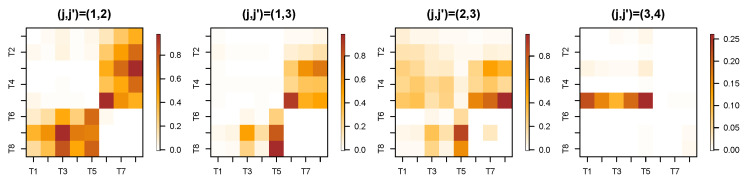
The *p*-values based on the permutation test for cross-scale coherence among the given pair of LFP in different trials of Superchris.

To investigate whether there are changes in brain connectivity when the rat makes mistakes compared to correct responses, we conducted a permutation test with 1000 replicates. This test aimed to identify significant differences in the average wavelet coherence between trials with correct and incorrect responses. Figure 17 presents the *p*-value results across channels and specific pairs of subprocesses, corresponding to the cross-scale coherence shown in Figure 16.

**Figure 18:**
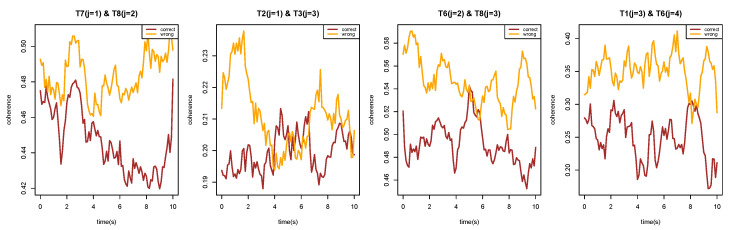
The average time-varying cross-scale wavelet coherence between the subprocess at specific channels and scales across corresponding trials.

Based on the results of the permutation test, we selected several pairs of scale-specific subprocesses from different channels that correspond to significant *p*-values in Figure 17. This selection aimed to verify whether the differences between correct and incorrect trials are clearly observable. Figure 18 demonstrates that the cross-scale coherence between these selected subprocesses shows significant differences across the two types of trials. Our framework effectively captures the time-evolving coherence, revealing that, in most cases, the coherence during incorrect trials is substantially higher than that observed in correct trials.

The analysis reveals numerous alterations in brain connectivity during incorrect responses, even in cross-scale interactions between different regions. These findings highlight the effectiveness of wavelet coherence as a powerful tool for capturing critical dynamics in brain activity.

## 7. Topological Data Analysis

As described in the previous sections, numerous methods have been proposed to estimate brain connectivity, spanning from correlation- and coherence-based measures to Granger causality, transfer entropy, and wavelet-based approaches for non-stationary data. The subsequent step typically involves performing a brain network analysis under different scenarios.

Brain network analysis has emerged as a vital area of research for understanding neural connectivity and its role in cognitive and physiological processes [5]. Over the past few decades, this field has been shaped by foundational studies in network science, such as the concepts of small-world networks [61] and scale-free networks [62], which highlighted the key organizational principles of brain networks. These studies have motivated the application of graph-theoretic approaches to analyze brain connectivity, providing valuable insights into the structural and functional organization of the brain [63,64].

Graph-theoretic measures, such as the clustering coefficient and modularity, have been extensively used in brain network analysis. The clustering coefficient quantifies the extent to which a node is connected to or influences its neighbors, providing insights into how different brain regions collaborate to process information. Modularity, in contrast, measures the extent to which a network can be divided into distinct communities or modules with dense intra-community connections and sparse inter-community links, offering a deeper understanding of functional segregation in the brain. Physiologically, these measures have been linked to cognitive processes such as information integration and function segregation [65,66].

Despite their utility, graph-theoretic approaches have notable limitations. One major challenge is the thresholding problem, where the process of binarizing or sparsifying (creating an adjacency matrix from edge weights) connectivity matrices can significantly affect results, introducing subjectivity and potential bias [67,68]. Additionally, measures like clustering coefficient and modularity are summaries of the graph and may overlook more intricate, multi-scale interactions beyond pairwise relationships within the brain network. This limitation has prompted the exploration of alternative methods that can capture the richer and more detailed features of connectivity.

Topological data analysis (TDA) methods have gained significant momentum in recent years, especially in the analysis of brain signals due to its ability to characterize the shape and structure of multivariate time series data across multiple scales [69,70]. One of the key tools in TDA, persistent homology (PH), has proven particularly powerful for understanding the topological structure of data. For example, Lee et al. [71] was among the first to introduce PH to brain network analysis, comparing functional networks across groups of children with ADHD, ASD, and typical development. Similarly, Wang et al. [72] applied PH to event-related potentials, successfully detecting differences between post-stroke aphasic individuals and healthy controls under conditions of altered auditory feedback. Additionally, Saggar et al. [73] demonstrated the utility of the Mapper algorithm for reducing the dimensionality of connectivity graphs, thereby facilitating the analysis of dynamic brain networks and task-related effects.

**Figure 19:**
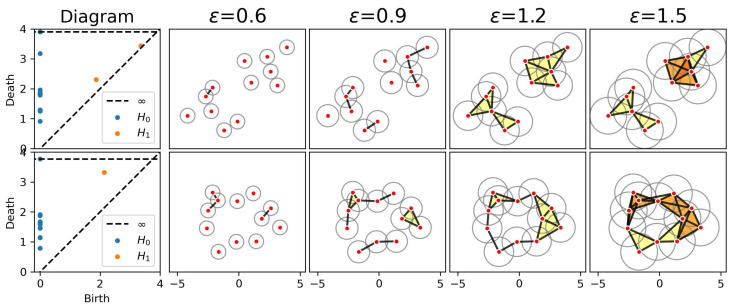
Two examples of Vietoris–Rips filtrations on point-cloud data, along with their corresponding persistence diagrams. The top example shows two distinct clusters, and the bottom example features a single cycle. The four columns on the right illustrate ball coverings (and their nerves) at increasing radii. Persistence diagrams plot birth–death pairs (b,d) for topological features, with different dimensions represented by distinct colors.

PH analyzes the evolution of topological features, such as connected components (clusters), loops (cycles), and higher-dimensional voids, across a scale parameter. As illustrated in Figure 19, PH constructs a filtration, that is, a nested sequence of simplicial complexes that extend the notion of networks beyond pairwise interactions. By tracking the “birth” and “death” of these features, PH reveals the scales at which significant topological structures emerge, offering a nuanced view of neural connectivity. The figure demonstrates this process with two examples: the top row represents a dataset with two distinct clusters, while the bottom row illustrates a dataset with a single prominent cycle. As the parameter ϵ increases (illustrated by growing balls around the data points in the four rightmost columns), the filtration encodes the topology at different scales. For the clusters, the features persist until they merge, while for the cycle, the loop appears at a certain scale and disappears at another. PH typically summarizes this information using visual tools such as barcodes [74], persistence landscapes [75], persistence images [76], or persistence diagrams as illustrated in the left column of Figure 19, where for each dimension, the birth–death pairs (b,d) of topological features are plotted as points in the (x,y)-coordinate system, with different dimensions represented by distinct colors. For instance, connected components ($H_{0}$) are shown as points on the y-axis (b=0), while cycles ($H_{1}$) are points in the upper triangle (b<d) with points farther away from the diagonal, indicating a longer persistence.

By tracking these features and representing them in diagrams, PH offers a deeper understanding of the underlying data structure. In the context of brain networks, it reveals connectivity patterns that extend beyond traditional graph-theoretic measures, providing a robust framework for studying neural dynamics and organization. For time series data, the Vietoris–Rips filtration can be constructed in various ways [70].

To analyze the connectivity patterns in the rat’s LFP data, we focus on in-sequence trials (A, B, C, D, E) where the rat made correct decisions recognizing the odor, resulting in a total of 190 trials. For each trial, we estimate coherence matrices across different frequency bands and construct the corresponding persistence diagrams (PDs).

**Figure 20:**
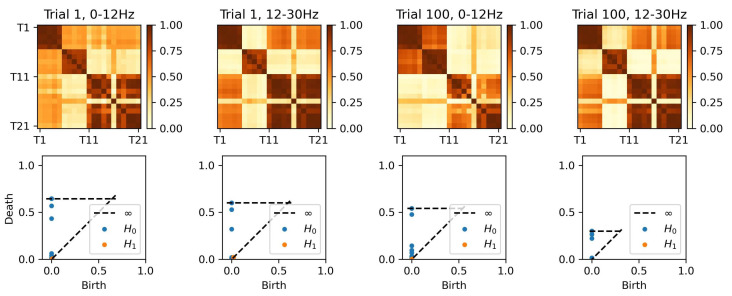
Persistence homology based on coherence matrices. The first row shows coherence matrices for Trial 1 (0–12 Hz, 12–30 Hz) and Trial 100 (0–12 Hz, 12–30 Hz), from left to right. The second row displays the corresponding persistence diagrams.

Figure 20 illustrates the results for two selected trials (Trial 1 for odor A and Trial 100 for odor B) at two frequency bands (0–12 Hz and 12–30 Hz). The first row displays the coherence matrices for each trial and frequency band, offering insights into the pairwise interactions between regions. The second row showcases the associated persistence diagrams, which summarize the birth and death times of the 0D and 1D topological features, providing a compact representation of the topological structure inherent in the coherence matrices.

All four coherence matrices appear to display similar information, with three main clusters visible across the trials. Within the rat hippocampus, the distal (first half of the tetrodes) and proximal (second half of the tetrodes) regions exhibit distinct patterns: two clusters are evident in the distal region, while only a single cluster is apparent in the proximal region. However, the visually distinguishing differences between the coherence matrices across trials and frequency bands remains challenging. In contrast, the PDs provide more precise information on the birth and death times of topological features, clearly highlighting differences between the trials that are not easily observable in the coherence matrices.

A persistence diagram $D_{k}$ for a dimension *k* is a multiset of birth–death pairs:

$$D_{k}=\left\{\left(b_{i},d_{i}\right)|b_{i},d_{i}\in \bar{R},\;b_{i}<d_{i}\right\},$$

where each $\left(b_{i},d_{i}\right)$ encodes the appearance ($b_{i}$) and disappearance ($d_{i}$) scales of a topological feature of dimension *k*.

This approach enables a rigorous comparison of the topological structures across trials, offering insights that surpass traditional graph-theoretic measures. By estimating dependence for each trial and applying PH, we assess the topological features in connectivity graphs. Using the Wasserstein distance (see Equation (17)), we quantify changes in connected components (dimension 0) and cycles (dimension 1) across trials, uncovering subtle differences in neural dynamics. This approach enables a rigorous comparison of the topological structures across trials, offering insights that surpass traditional graph-theoretic measures. By estimating dependence for each trial and applying PH, we assess the topological features in connectivity graphs. Using the Wasserstein distance (see Equation (17)), we quantify changes in connected components (dimension 0) and cycles (dimension 1) across trials, uncovering subtle differences in the neural dynamics:

(17) $$d_{W}\left(D_{1},D_{2}\right)=\min_{\Gamma }{\left(\;\sum_{(x,y)\in \Gamma }{∥\;x-y\;∥}_{\infty }^{2}\right)}^{\;1/2},$$

where Γ ranges over all bijective matchings between points in $D_{1}$ and points in $D_{2}$ (possibly adding diagonal points if needed).

**Figure 21:**
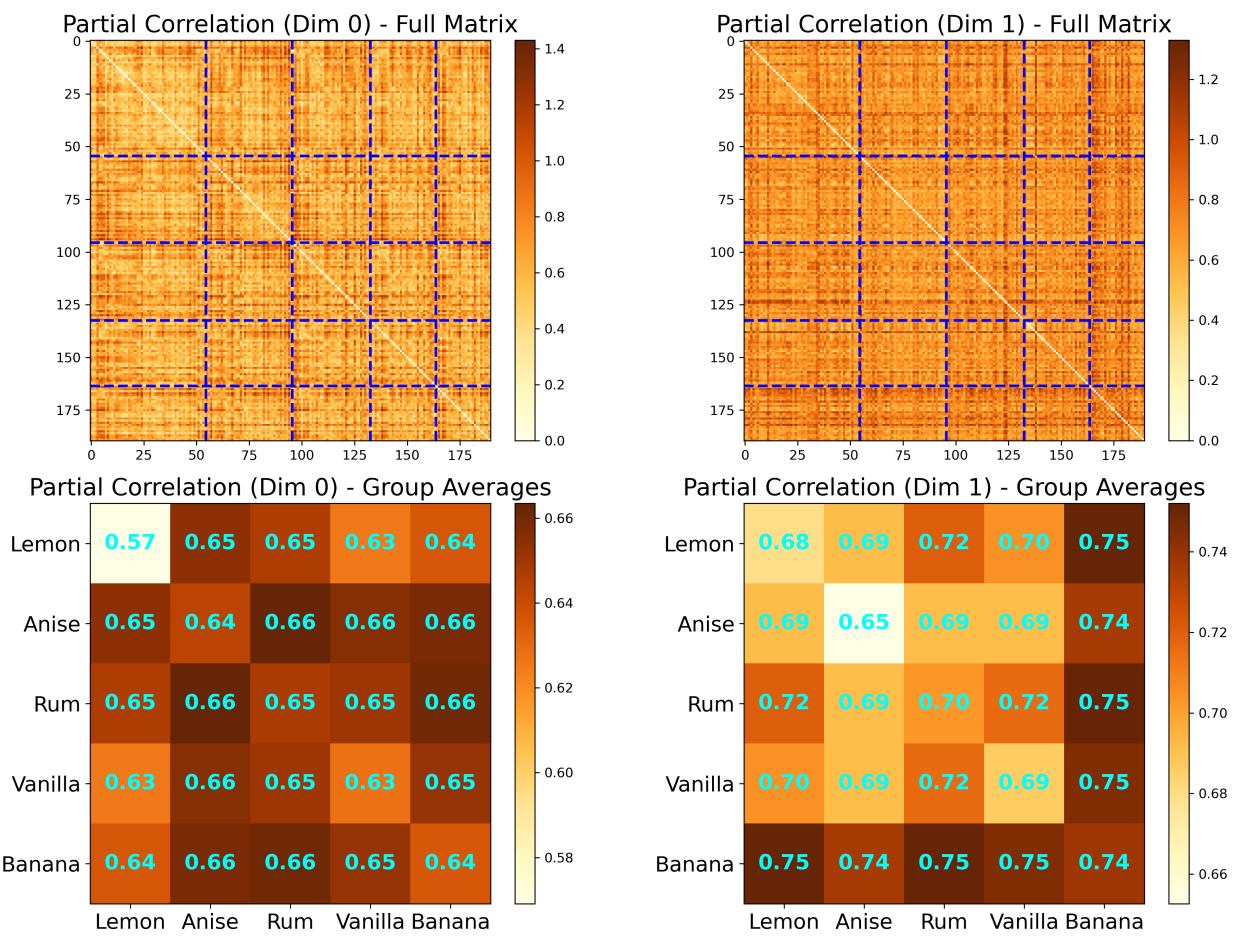
Topological analysis of partial correlation dependence. The top row presents the full Wasserstein distance matrices for dimensions 0 (**left**) and 1 (**right**). The bottom row displays averages over trials for each odor, illustrating variability in connected components and cycles across odors. For dimension 0, the first odor (lemon) shows reduced variability, while dimension 1 highlights minimal changes for the second odor (anise).

To analyze the partial correlation dependence, we compute the Wasserstein distances between persistence diagrams across trials. The results are presented in Figure 21, where the top row shows the full distance matrices for dimensions 0 (left) and 1 (right), and the bottom row summarizes averages grouped by odors (lemon, anise, rum, vanilla, and banana). For dimension 0, the variability in connected components is notably smaller for trials corresponding to the first odor (lemon) compared to the others. Conversely, for dimension 1, the second odor (anise) shows the lowest variability, suggesting distinct patterns in topological features depending on the odor and dimension.

**Figure 22:**
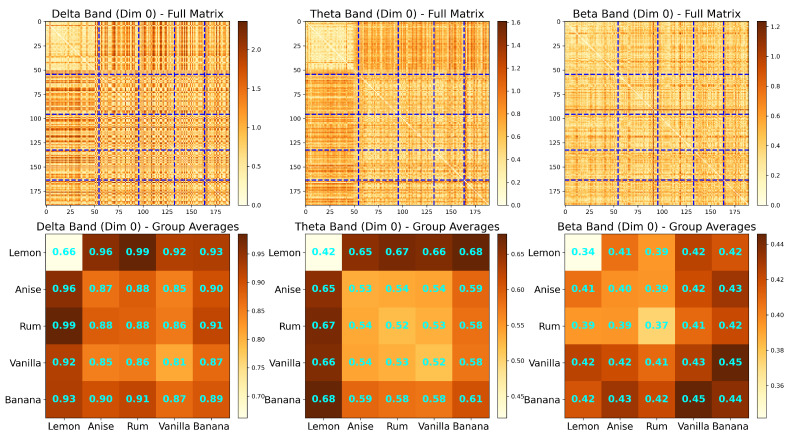
**Low-frequency range (0–12 Hz).** Wasserstein distance matrices for coherence-based analysis in dimension 0 across three frequency bands: delta, theta, and beta (**left** to **right**). The top row shows full matrices, while the bottom row presents averages over trials for each odor. The lemon odor exhibits lower variability in the delta and theta bands but not in the beta band.

**Figure 23:**
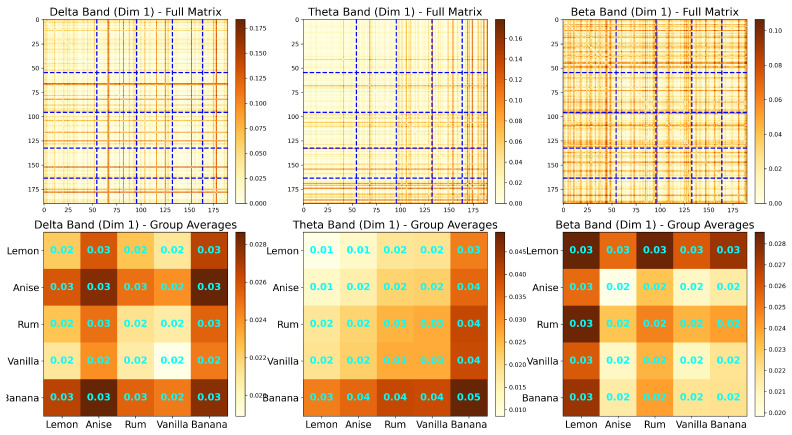
**Medium-frequency range (12–30 Hz).** Wasserstein distance matrices for coherence-based analysis in dimension 1 across three frequency bands: delta, theta, and beta (**left** to **right**). Similar to dimension 0, the top row shows full matrices, and the bottom row provides averages over trials for each odor. Changes in cyclic patterns appear relatively consistent across odors and frequency bands.

The coherence-based analysis highlights frequency-specific topological patterns across trials and odors. Figure 22 focuses on dimension 0, showing that the lemon odor exhibits lower variability in connected components within the delta and theta bands but not in the beta band. This indicates that distinct frequency bands capture different aspects of connectivity. Meanwhile, Figure 23 explores cycles (dimension 1) across the same frequency bands, showing relatively consistent patterns with no strong odor-specific differences.

By combining these results, we highlight the value of PH in identifying nuanced patterns in brain connectivity networks. The coherence-based analysis underscores the ability to pinpoint frequency bands where significant topological changes occur, while the partial correlation analysis reveals odor-specific differences in neural connectivity. Together, these findings demonstrate the potential of persistent homology to provide a deeper understanding of the organization and dynamics of complex neural systems.

In summary, one of the key strengths of TDA lies in its ability to move beyond pairwise interactions and capture higher-order structures in complex networks. While many traditional graph-based tools focus on edges between pairs of nodes, TDA leverages simplicial complexes to incorporate multi-node (higher-order) dependencies. In parallel, new frameworks have emerged to integrate higher-order interactions into measures of dependence such as transfer entropy [77], and recent investigations of brain connectivity underscore the importance of these high-order relationships [78,79]. Advanced TDA approaches like Hodge decomposition have extended standard methodologies (e.g., persistent homology) from handling only symmetric connectivity to accommodating non-symmetric dependency measures [80]. This expansion enables TDA to capture global topological patterns (gradient, local, and global loops) when the underlying networks arise from effective connectivity. The ability to incorporate such higher-order interactions in non-symmetric, directed settings highlights a promising avenue for future research.

## 8. Discussion

As fundamental measures of linear association, correlation and coherence have been extensively utilized to assess functional connectivity in neuroscience research. Coherence, in particular, offers a more nuanced analysis when dependence is driven by specific oscillations, forming the foundational elements of most brain connectivity analyses.

Building upon these principles, KenCoh has been developed to address some of the limitations inherent in coherence. Specifically, it enhances the ability to discern more complex patterns of connectivity that are not readily apparent with traditional coherence measures. Moreover, it provides region-to-region analyses that align with the spatial orientation of most brain imaging data. In Section 3, we apply KenCoh to LFP data to investigate Buchanan’s brain connectivity during in-sequence and out-of-sequence trials. The results indicate that the same key tetrodes contribute more to global coherence in the beta band during in-sequence trials, contrasting with the findings from out-of-sequence trials. This suggests a potential role in the pattern recognition skills of rats.

Granger causality analysis is designed to capture directional interactions between brain regions, offering deeper insights into effective connectivity. Unlike functional connectivity which reflects statistical associations without implying causation, GC indicates the direction of information flow between regions. This directional information is especially valuable for studying specific pathways, such as those involved in sensory integration or memory formation, without needing to map the entire connectivity structure. Moreover, when combined with sDPCA, GC preserves the dominant oscillatory activity in the broader network before focusing on pairwise interactions, thereby enhancing the reliability of inferred direct influences.

Pairwise GC analysis of the LFP recordings indicate a dominant flow from distal to proximal CA1 across odors, although certain subjects show additional reverse or two-way interactions. One subject exhibits extensive reciprocal connectivity, contrasting the more unilateral patterns observed in others. These findings suggest that hippocampal olfactory processing depends on a shared distal-to-proximal route, with odor-specific link changes superimposed, reflecting subtle variations in how each subject’s CA1 circuitry responds to different odor conditions.

One major advantage of the STE method in analyzing LFP data is that it enables the capturing of nonlinear (possibly cross-frequency) information transfer between nodes in a brain network, with minimal assumptions on the distribution or type of relationship between the signals. That is, it allows for quantifying effective brain connectivity that concentrates on specific frequency bands, which makes it straightforward to link results to well-established findings in cognitive neuroscience. Also, its application to understanding effective brain connectivity is not limited only to LFP data but to other brain imaging modalities such as EEG and functional near infrared spectroscopy (fNIRS). Moreover, its estimation is simple and computationally efficient, as it employs a vine copula approach as illustrated in [52]. Since STE is defined over maximum amplitudes of non-overlapping time blocks, it is fairly robust to spontaneous noise artifacts which may primarily take effect at high-frequency oscillations. These advantages enable us to identify major differences in the magnitude of information flow between the distal and proximal regions of the subjects during in-sequence and out-of-sequence trials in the alpha and beta frequency bands.

A caveat, however, is that the STE approach assumes the stationarity of signals because it requires the extraction of band-specific oscillations through bandpass filtering (e.g., Butterworth filter). The stationarity assumption ensures the extracted signals appropriately capture the oscillations of interest. In our LFP analysis, this is not an issue since the 1.2 s segments we analyze exhibit quasi-stationary behavior. In addition, the temporal resolution of causality captured by STE is relatively slower than the actual sampling rate of the signal due to the aggregation over time blocks. Depending on the choice of block size *m*, the causal interpretations for the connections measured by STE change. Thus, practical considerations, aligning with the goals of the study, should be made before implementation to achieve its best performance.

Often brain signals are assumed to be stationary. However, this is not the case in many practical scenarios. Wavelet coherence addresses these challenges effectively by analyzing non-stationary time series and capturing time-varying statistical properties within these signals. The application of wavelet coherence analysis to LFP data helps us identify intriguing interactions between components at different scales across various channels. Furthermore, we observe distinct differences among channels when the rat makes mistakes compared to correct responses, providing valuable insights into the neural dynamics.

Persistent homology provides a robust framework for analyzing brain connectivity in LFP recordings by quantitatively assessing the shape and structure of high-dimensional brain networks. This capability is particularly valuable for investigating how different tasks or conditions affect brain organization and for comparing individuals with varying neurological disorders. Our analysis demonstrates that TDA can reveal subtle, yet meaningful, variations in neural connectivity that conventional methods often overlook. Specifically, persistence diagrams derived from both coherence and partial correlation matrices highlight the variations that are both odor specific and frequency specific. For instance, in the partial correlation analysis, the persistence diagrams show that trials associated with the lemon odor exhibit reduced variability in connected components (dimension 0), suggesting a more stable clustering of neural activity. In contrast, trials linked to the anise odor display lower variability in cyclic features (dimension 1), indicating more consistent loop structures. Similarly, the coherence-based analysis reveal that low-frequency bands (delta and theta) capture more stable connectivity patterns for the lemon odor compared to the beta band.

### 8.1. Advantages and Limitations

The presented methods present distinct strengths and limitations in capturing the various aspects of neural connectivity. To summarize these findings and aid in method selection, Table 2 presents a comprehensive comparison of the advantages and limitations of each approach, addressing the specific challenges inherent in neural data analysis.

### 8.2. Future Directions

The theoretical properties of KenCoh remain an open question and warrant further investigation. In particular, it would be interesting to examine the performance of KenCoh when the group sizes of variables become large, i.e., as *P* and *Q* tend to infinity. A natural approach to addressing the high dimensionality of the problem is to regularize the canonical directions u and v. For instance, one could impose additional constraints, such as ${\left|u\right|}_{L_{1}}={\left|v\right|}_{L_{1}}=1$, to obtain a sparse solution while solving the maximization problem in (10).

In the relatively novel field of neural network-based Granger causality (NN-GC), we see an extension of traditional causality concepts. Conventional approaches assume a linear dependence structure in data or provide hand-selected basis functions or kernel transformations which requires domain knowledge and expertise. NN-GC approaches leverage the function approximation power of neural networks to model complex, nonlinear interactions that are not easily captured by standard statistical methods. These methods relax the linearity assumption of standard methods and learns data-driven features in an end-to-end manner through error backpropagation. Several methods based on NN have been recently proposed based on sparse regression. We refer the interested reader to [81,82,83,84] for more details.

Deep learning-based approaches to GC discovery from observational time series data have considerable potential due to the neural network models’ ability to learn task-specific, data-driven representations. Although sparse regression-based techniques have been proposed in the literature [81,82,83,84], these methods do not provide uncertainty quantification in their estimates. In addition, developing efficient Auto-ML and sensitivity analysis techniques to optimize hyperparameters (e.g., regularization, sparsity, and optimizer settings) and to reduce computational cost, as well as exploring time-varying and multi-scale GC analyses (where neuronal states can switch between connectivity patterns over different frequency bands or behavioral conditions), represent further directions to enhance these methods.

In its current formulation, STE addresses effective connectivity between nodes in a brain network in a pairwise manner. That is, it does not account for how other parts of the network, say signals from a third node, affect the strength of information transfer between the pair of nodes being investigated. Thus, one interest extension is to develop a new metric based on causation entropy, which is another information-theoretic measure, that captures the magnitude and direction of information flow between two variables after taking into account the contributions of other variables in the system.

The definition of wavelet coherence can be extended to address more complex scenarios, such as locally stationary partial coherence in the presence of high-dimensional confounders. This extension would further capitalize on the time–frequency localization capabilities of wavelets, offering improved sensitivity in detecting dynamic connectivity patterns.

While most TDA techniques focus on functional connectivity, Hodge decomposition, a method rooted in algebraic topology offers a complementary perspective on effective connectivity. By decomposing brain connectivity into gradient, curl, and harmonic components, this approach can reveal subtle dynamics in the flow of information that are often disrupted in neurological disorders [80]. Furthermore, integrating this decomposition with machine learning techniques holds promise for detecting abnormal connectivity patterns associated with specific conditions, potentially paving the way for improved diagnostics and targeted therapies (e.g., in epilepsy).

## 9. Conclusions

In this paper, we analyzed brain connectivity data from the hippocampal region of rats using a diverse set of methods. Our approach spanned traditional techniques such as correlation, partial correlation, and coherence—and advanced methods including Granger causality, robust canonical coherence, spectral transfer entropy, wavelet coherence, and persistent homology. By comparing these techniques, we provided a detailed examination of their strengths, limitations, and their applicability to uncovering the complex interactions within neural systems.

Our findings demonstrate that classical methods serve as a reliable foundation for capturing linear and stationary relationships, while advanced techniques are better suited to capture nonlinear, dynamic, multi-scale and higher-order interactions.

The application of these methods to hippocampal LFP data revealed nuanced, odor-specific, and frequency-specific patterns in connectivity, which underscore the complex organization of neural circuits underlying nonspatial olfactory processing. Despite these promising results, several challenges remain, including the need for careful parameter tuning, computational efficiency, and improved interpretability of some of these advanced techniques.

Integrating these diverse methods into unified frameworks that leverage their complementary strengths could offer even deeper insights into brain connectivity. Moreover, the development of scalable algorithms and user-friendly software tools is essential for translating these advanced techniques into practical applications for neuroscience research.

By presenting a comprehensive suite of methods and applying them to hippocampal LFP data, this study aims to pave the way for further exploration and innovation in brain connectivity analysis. 

## Figures and Tables

**Table 1:** Details of the trials conducted on the five subjects.

Subject	No. of Tetrodes	In-Sequence		Out-of-Sequence	
		Correct	Incorrect	Correct	Incorrect
Barat	22	154	1	11	10
Buchanan	20	203	23	29	15
Mitt	22	230	32	16	14
Stella	21	176	18	23	5
Superchris	21	190	20	26	4

**Table 2:** Comparative summary of methods for brain connectivity analysis.

Method	Advantages	Limitations
**KenCoh**	Looks beyond pairwise association. Robust to outliers. The estimator has closed-form expression, unlike its robust alternatives. Computationally efficient.	Assumes stationarity of the time series. The vector of random amplitudes is assumed to have elliptic density. The components of the vector of random amplitudes are assumed to have equal variances.
**sDPCA-GC**	Preserves key oscillatory patterns via frequency-aware reduction. Permits standard GC for interactions between nodes-of-interest. Straightforward to implement and interpret once components are derived. Robust to moderate noise.	Selecting the number of principal components is non-trivial. Assumes linearity and stationarity. Physiological interpretation of dynamic principal scores is not straightforward.
**STE**	Captures nonlinear information transfer with minimal assumptions. Straightforward to link results to cognitive neuroscience. Simple and computationally efficient estimation. Robust to spontaneous noise artifacts.	Assumes stationarity of signals. Temporal resolution of causality is limited by the aggregation over time blocks.
**WaveletCoh**	Maintains time and scale information. Effective for capturing time-varying statistical properties within non-stationary time series.	Scale does not perfectly correspond to specific frequency bands, making interpretations challenging.
**TDA-PH**	Avoids thresholding weighted networks. Ability to identify complex topological patterns. Considers higher-order interactions. Robust to moderate noise.	Computationally intensive, especially for large networks with thousands of nodes. Global level results that can be hard to interpret. Sensitive to outliers. Cannot handle directed networks.

## Data Availability

The data is not available publicly. Researchers interested in accessing the data can contact Norbert J. Fortin at norbert.fortin@uci.edu.

## Abbreviations

The following abbreviations are used in this manuscript:

EEG	Electroencephalogram
ECoG	Electrocorticogram
LFP	Local Field Potentials
fMRI	functional Magnetic Resonance Imaging
CBC	Canonical Band-Coherence
MCD	Minimum Covariance Determinant
SMOTE	Synthetic Minority Over-sampling Technique
PCA	Principal Component Analysis
sDPCA	Spectral Dynamic Principal Component Analysis
VAR	Vector Autoregressive
GC	Granger Causality
NN	Neural Networks
NOI	Node of Interest
TE	Transfer Entropy
STE	Spectral Transfer Entropy
LSW	Locally Stationary Wavelet
MvLSW	Multivariate Locally Stationary Wavelet
TDA	Topological Data Analysis
PH	Persistence Homology
PD	Persistence Diagram
ADHD	Attention Deficit Hyperactivity Disorder
ASD	Autism Spectrum Disorder
